# Supplementary material for: Ce-mediated molecular tailoring on gigantic polyoxometalate {Mo132} into half-closed {Ce11Mo96} for high proton conduction
Source: Nat Commun. 2023 Aug 18;14:5025. doi: 10.1038/s41467-023-40685-0 (PMC10439156; doi:10.1038/s41467-023-40685-0)
Supplement: Supplementary file 1 — Supplementary Information [file 41467_2023_40685_MOESM1_ESM.pdf]

Supplementary Information

Ce-Mediated Molecular Tailoring on Gigantic Polyoxometalate $\{Mo_{132}\}$ into Half-Closed $\{Ce_{11}Mo_{96}\}$ for High Proton Conduction

Li et al.

Supplementary Methods

All reagents and solvents were purchased from commercial sources and used as received. IR spectra were recorded on an Alpha Centauri FTIR spectrophotometer on pressed KBr pellets in the range 400~4000 cm^{-1}. Elemental analysis of cerium and molybdenum were performed with a Leaman inductively coupled plasma (ICP) spectrometer; Carbon, sulfur and hydrogen content were determined by a Euro Vector EA3000 elemental analyzer. Water contents were determined by TG analyses on a PerkinElmer TGA7 instrument in flowing N_2 with a heating rate of 10 °C min^{-1}. A Rigaku D/max-IIB X-ray diffractometer was applied for the XRD characterizations (scanning rate: 5° min^{-1} with $Cu_{K\alpha}$ radiation, λ = 1.5418 Å). UV−Vis spectra were obtained by using a 752 PC UV/Vis spectrophotometer. XPS was performed on a ESCALAB 250 spectrometer. The vacuum inside the analysis chamber was maintained at 6.2×10^{-6} Pa during the analysis. The morphology of crystal was characterized with SEM (FESEM; XL30, FEG, FEI Company). Raman spectra were conducted by a Jobin Yvon confocal laser Raman spectroscopy. The Mott–Schottky spots were carried out at ambient environment using the electrochemical workstation (CHI 760E) in a standard three-electrode system at frequencies of 800, 1000 and 1200 Hz. The bulk proton conductivity was calculated by fitting the Nyquist plot with an electrical equivalent circuit below, which consists of a contact resistor (R1), bulk resistor (R2), capacitance (CPE1), and Warburg diffusion element (W1).

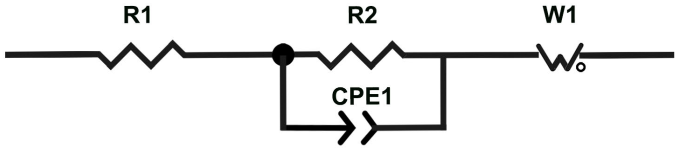

Supplementary Discussion

Synthesis discussion of **1**: The capped wheel {Mo_{132}} was synthesized from a one-pot reaction of an acidified aqueous mixture of $Na_2MoO_4\cdot2H_2O$ (0.72 mmol), $Na_2S_2O_4$ (0.60 mmol) and $CH_3COONa\cdot3H_2O$ (0.70 mmol) heated in a 25ml Teflon-lined stainless-steel container at 150 °C. The combined reagents changed from a colorless mixture to transparent, blue solution before heating. The key variables for the formation of capped wheel {Mo_{132}} were found to be both accurate pH tuning and constant high temperature. On one hand, high-nuclearity wheel-shaped molybdenum oxide clusters are commonly synthesized at a specific low pH value, see *Chem. Soc. Rev.* **41**, 7431-7463(2012). Thus, we analyze the formation of capped wheel {Mo_{132}} with pH value as the single variable: we found that increasing the pH value resulted in the oxidation of the Mo^{V} centers (white precipitate was formed), whilst much lower pH (pH < 1.0) yielded only amorphous precipitate. pH 1.8-2.5 is suitable for the assembly of capped wheel {Mo_{132}} with its highest yield located at pH 2.2. On the other hand, previously reported high-nuclearity wheel-shaped molybdenum oxide clusters had been synthesized from reactions heated to between 20~90 °C. By heating the reactant to 150 °C we bring the system from standard reaction conditions to hydrothermal reaction conditions. Hydrothermal method was conducted in a high-temperature and high-pressure state, see *J. Am. Chem. Soc.* **142**, 13982−13988(2020), which is good for the construction of new molybdenum oxide clusters featuring novel building blocks or special configuration (*e.g.* capped wheel molybdenum oxide cluster). The high temperatures between 140 and 155 °C are suitable for the formation of capped wheel {Mo_{132}} with its highest yield located at 150 °C. The results showed that hydrothermal reactions could open a whole new world to the chemistry of giant polyoxomolybdates, which had been almost exclusively prepared under ambient conditions. Besides,

capped wheel $\{Mo_{132}\}$ could be obtained after quadrupling the amount of reactants with the yield remains little change.

Synthesis discussion of **2**: During the synthesis, two important factors should be emphasized: (1) the choice of Ce ions. 4*f* lanthanide (Ln) ions are oxophilic and display high coordination numbers as well as relatively long Ln-O bond lengths, which are usually used as strong electrophiles to replace edge-sharing or corner-sharing $\{Mo_2\}$ units on the parent polyoxomolybdate framework. For example, Ln^{III} center with the coordination configuration of a distorted monocapped square antiprism, built from four μ_2-O atoms and five H_2O molecules − $\{Ln(H_2O)_5\}$, joins two sets of $\{Mo_6\}$ with four Mo−O−Ce bonds, see *J. Am. Chem. Soc.* **136**, 14114−14120(2014), in a manner similar to a $\{Mo_2\}$ unit (both act as 4-connected rods). Such coordination configuration remains the key point why lanthanide ions are easy to be incorporated into polyoxomolybdate wheels. In all, we choose lanthanide ions to dissociate the Mo ball mainly due to its high coordination number (nine) and flexible coordination environment ($\{Ln(H_2O)_5\}$, distorted monocapped square antiprism). In fact, during the preparation of $\{Ce_{11}Mo_{96}\}$, all 4*f* lanthanide ions (except radioelement) were introduced into the reaction system for exploring the Ln-mediated molecule tailoring process. When lanthanide ion was absent, no crystals but precursor $\{Mo_{132}\}$ was obtained. Simple 3*d* metal salts (*e.g.* Co^{2+}, Mn^{2+}, Fe^{3+}, *etc.*) or metal coordination complexes (*e.g.* $\{Mn_{12}\}$, Ni complex $[Ni_2(\mu\text{-}OH_2)(O_2CCMe_3)_4(HO_2CCMe_3)_4]$, Cr complex $[Cr_3O(OOCCH_2CN)_6(H_2O)_3]^+$, *etc.*) were also introduced to the acid solution, but no crystals was obtained. We found that only 4*f* lanthanide La, Ce and Pr ions could be harvested with crystal samples. However, La- or Pr-containing samples possess rather poor crystal quality that the data are really hard to be collected although careful optimization related to the reaction conditions had been conducted. So we just report the Ce-containing samples with good crystal quality for publication. At this level, previous reports show that the Ln-containing isomers in POMs possess similar IR spectra for polyoxometalate “fingerprint region”, see *Dalton Trans.* 4423–4425(2009), *Chem. Eur. J.* **20**, 12144–12156(2014), *Chem. Eur. J.* **21**, 18168–18176(2015). As show in Supplementary Figure 1, the current La-, Ce- and Pr-containing samples possess similar IR spectra for polyoxometalate “fingerprint region”. So we guess that Ln-mediated molecule tailoring process could be realized through La, Ce and Pr ions. The lanthanide contraction results in smaller ionic radii as we cross the 4*f* period which becomes a key factor in the replacement of $\{Mo_2\}$ from Pr onward. This phenomenon is common in Ln-containing giant Mo-oxo clusters, see Supplementary Table 1. We found that the 4*f* lanthanide ions are mainly focus on early lanthanide ions or just Ce ions.

(2) the use of additional SO_4^{2-}. When additional Na_2SO_4 was introduced into this reaction system, the $\{Ce_{11}Mo_{96}\}$ product could be obtained with a relatively high yield (~25%). The yield without Na_2SO_4 is 16%. Comparative experiments showed that the yield keeps unchanged when Na_2SO_4 was replaced by NaCl or $CH_3COONa\cdot 3H_2O$, which we found that the ionic strength of sulfite anion in the reaction solution possesses certain influence on the synthesis. In general, the sulfite anion as inorganic ligand introduces the necessary diversity into POM systems, see *Inorg. Chem.* **53**, 9486−9497(2014); *Angew. Chem. Int. Ed.* **42**, 2085–2090(2003); *Angew. Chem. Int. Ed.* **47**, 8420–842(2008). This is in line with the structural analysis, the SO_4^{2-} are important factors during the assembly of $\{Ce_{11}Mo_{96}\}$. Sulfite anion inside half-closed $\{Ce_{11}Mo_{96}\}$ provides a suitable microenvironment for trapping ‘inner’ cerium centers, which plays a key role in the formation of Ce-containing product $\{Ce_{11}Mo_{96}\}$.

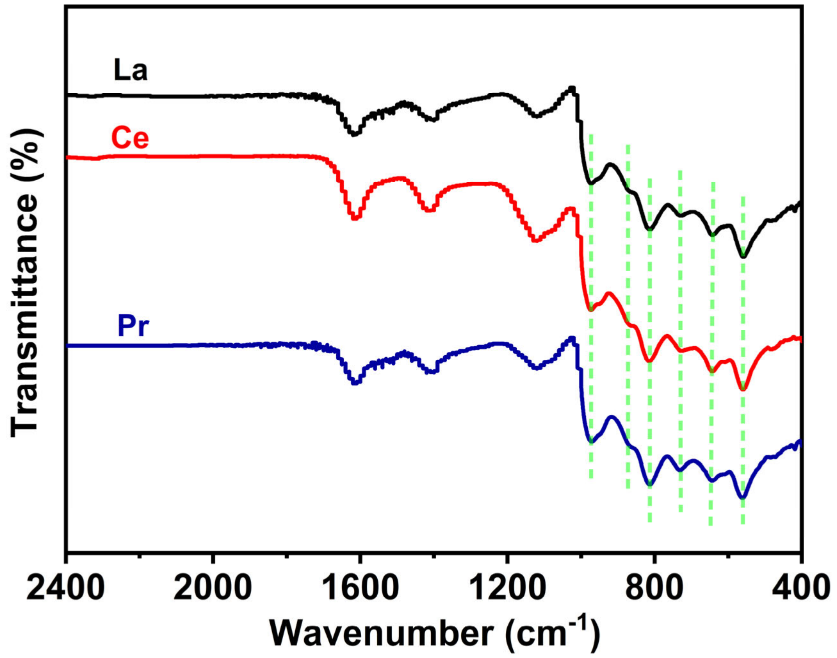

Supplementary Figure 1. IR spectra for {$Ln_{11}Mo_{96}$} (Ln = La, Ce or Pr). IR spectra for polyoxometalate "fingerprint region" of La-, Ce- or Pr-containing samples.

Supplementary Table 1. Some reported representative Ln-containing giant Mo-oxo clusters.

Year	Compounds	lanthanide ions	*Ref.*
2022	$[Mo_{64}Ni_8Ln_6H_{26}O_{200}(H_2O)_{30}]^{8-}$	La, Ce, Pr	*Angew. Chem. Int. Ed.* e202201672 (2022)
2021	$[Mo_{124}Ce_4O_{376}(H_2O)_{64}H_{12}(C_8H_{13}N_4O_3)_4]^{3-}$	Ce	*Chem. Sci.* **12**, 2427–2432(2021)
2020	$[H_{16}Mo_{124}Ce_4O_{376}(H_2O)_{56}(PMo_{12}O_{40})(C_6H_{12}N_2O_4S_2)_4]^{5-}$	Ce	*Proc. Natl. Acad. Sci* **117**, 10699–10705(2020)
2019	$\{(Mo_8O_{26})Mo_{124}Ce_4O_{376}(H_2O)_{60}H_{12}(C_6H_9N_3O_2)_6\}^{12-}$ $\{Mo_{124}Ce_4O_{376}(H_2O)_{60}H_{12}(C_6H_{14}N_4O_2)_6\}^{8-}$	Ce	*J. Am. Chem. Soc.* **141**, 1242−1250(2019)
2019	$[H_{12}Mo_{128}Ce_4O_{389}(H_2O)_{60}(C_5H_{13}N_2O_2)_6]^{4-}$ $[H_{14}Mo_{158.5}Ce_2O_{478}(H_2O)_{81}(C_5H_{13}N_2O_2)_6]^{5-}$	Ce	*Angew. Chem. Int. Ed.* **58**, 10867–10872(2019)
2017	$Ce_{0.5}[Ce_6H_{16.5}Mo_{130}O_{396}(H_2O)_{84}]$	Ce	*Angew. Chem. Int. Ed.* **56**, 9727–9731(2017)
2014	$[Mo_{100}Ce_6O_{306}H_{10}(H_2O)_{71}]_2^{8-}$ $[Mo_{100}Ce_6O_{306}H_{10}(H_2O)_{70}]^{4-}$	Ce	*J. Am. Chem. Soc.* **136**, 14114−14120(2014)
2010	$[Mo_{96}O_{301}(H_2O)_{29}\{La(H_2O)_6\}_2]\{La(H_2O)_5\}_6]^{22-}$	La	*Inorg. Chem.* **49**, 9426–9437(2010)
2006	$[Mo_{150}O_{452}H_2(H_2O)_{66}\{La(H_2O)_5\}_2]^{24-}$	La	*J. Alloys Compd.* **408**, 693–700(2006)
2002	$[\{Mo_{128}Eu_4O_{388}H_{10}(H_2O)_{81}\}_2]^{20-}$	Eu	*Angew. Chem. Int. Ed.* **41**, 2805-2808(2002)
2000	$[Mo_{120}O_{366}(H_2O)_{48}H_{12}\{Pr(H_2O)_5\}_6]^{6-}$	Pr	*Inorg. Chem.* **39**, 3112-3113(2000)

Formula Determination

Structural analysis of **1** and **2**

Although the molybdenum cluster architectures are very complex, the general approach to the structural analysis and formula determination is well documented based on giant wheel- and ball-shaped polyoxomolybdates (*Acc. Chem. Res.* **33**, 2-10(2000), *J. Am. Chem. Soc.* **142**, 13982−13988(2020), *J. Am. Chem. Soc.* **142**, 17508−17514(2020)). The structural analysis requires the following lines of evidence/information to allow the assignment of formula and the structural details coupled with Single-crystal X-ray diffraction:

(i) Redox titration to help determine the number of reduced Mo^{V} centres.

(ii) Bond valence sum analysis to confirm the terminal oxo positions, reduced Mo^{V} centres and the positions of the hydroxide ligands.

(iii) Elemental analysis of molybdenum and cerium analysis.

(iv) TGA to estimate the solvent water molecules.

Therefore, the analysis below both presents this data and demonstrates how the structural assignment is consistent with this data.

Redox titrations

The cerimetric titration was carried out using a 0.005 M solution of Ce^{IV} in 0.5 M of sulphuric acid as oxidant which was added dropwise to a solution of **1** (20 mg in 50 mL of H_2O). After addition of 10.12 mL of the oxidant the colour of the solution turned from deep green to colourless along with a characteristic potential jump showed the presence of 60 ± 1 4d electrons which (formally) corresponds to 60 Mo^{V} centres (theoretical value for 60 e^{-} reduced species: 10.20 mL). As for **2**, it is impossible to perform redox tritration to determine the reduced eletrons on it due to its rather poor solubility in water as reported in previous Ce-containing molybdenum wheels literatures, see *J. Am. Chem. Soc.* **141**, 1242−1250(2019); *J. Am. Chem. Soc.* **142**, 17508−17514(2019). The Uv-vis spectra of **2** (Supplementary Figure 2) shows the characteristic band of Mo Green (centered at ca. 730 nm), which is similar to the characteristic spectral absorption of **1** (ca. 720 nm) and also different from the characteristic absorption of Mo Blue $\{Mo_{154}\}$. This comparison points that Mo Green **1** and **2** contain both 20 reduction electrons delocalized over their main wheel.

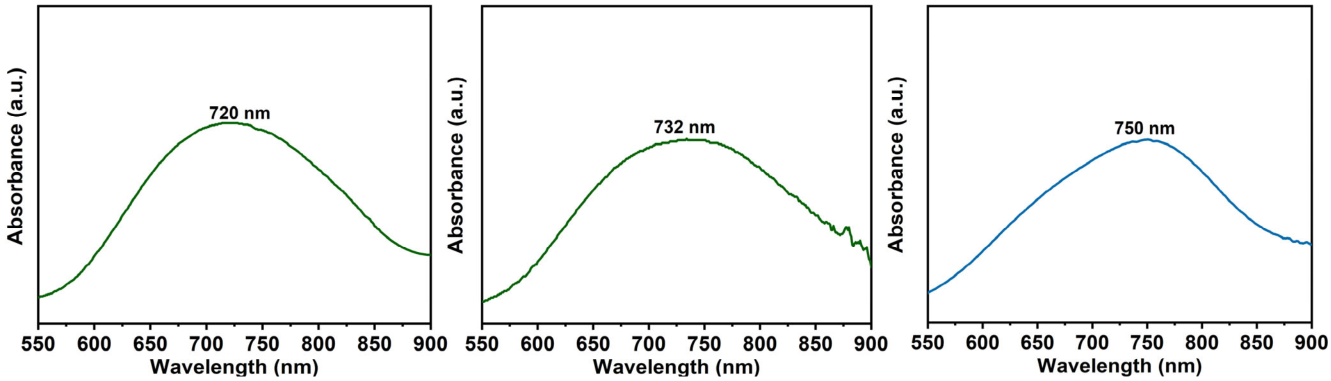

Supplementary Figure 2. UV-vis spectra. UV-vis spectra of **1** (left), **2** (middle) and $\{Mo_{154}\}$ (right).

Bond valence sum analysis

Supplementary Table 2. Average Bond valence sum values for the Mo centers in {Mo_4O_6}/{Mo_5O_6}-type double cubanes and the μ_3-O atoms of the {(μ_3-O)$_2$O$_2$}-type compartments in **1** and **2**.

Compounds	BVS (Mo)	BVS (μ_3-O)
**1**	5.64	1.35
**2**	5.69	1.60

Supplementary Table 3. Bond valence sum values for the edge-shared {Mo_2} units in {Mo_{110}} wheel of **1**.

Atom	Bond Valence	Atom	Bond Valence
Mo_{11}	4.88	Mo_{13}	5.04
Mo_{18}	5.12	Mo_{25}	4.96
Mo_{28}	4.94		

Supplementary Table 4. Bond valence sum values for {Mo_1} (between wheel and cap) in {Mo_8} of **1** and **2**.

Compouds	Atom	Bond Valence	Atom	Bond Valence	Atom	Bond Valence
**1**	Mo_{29}	5.16	Mo_{32}	5.19	Mo_{34}	5.29
**2**	Mo_{17}	5.08	Mo_{39}	5.04	Mo_{52}	5.07

Supplementary Table 5. Bond valence sum values of Mo^{V} in the $\{Mo_{11}\}$ caps of **1** and **2**.

Compouds	Atom	Bond Valence	Atom	Bond Valence
**1**	Mo_{26}	5.05	Mo_{31}	4.79
	Mo_{33}	4.93		
**2**	Mo_{18}	5.06	Mo_{38}	5.05
	Mo_{56}	4.93		

Supplementary Table 6. Bond valence sum values of the oxygen atoms for $\{Mo_{11}\}$ caps in **1** and **2**.

Compouds	Atom	Bond Valence	Atom	Bond Valence
**1**	O_{102}	0.27	O_{110}	0.25
	O_{116}	0.25	O_{118}	0.24
**2**	O_{12}	0.25	O_{82}	0.25
	O_{94}	0.26	O_{133}	0.33

Supplementary Table 7. Bond valence sum values of doubly protonated oxygen atoms in **2**.

	Atom	Bond Valence	Atom	Bond Valence	Atom	Bond Valence
terminal O atoms in $\{Mo_8\}$	O_{11}	0.27	O_{20}	0.26	O_{23}	0.28
	O_{40}	0.24	O_{63}	0.39	O_{75}	0.29
	O_{83}	0.31	O_{91}	0.26	O_{123}	0.25
	O_{141}	0.26	O_{149}	0.33	O_{159}	0.29
	O_{160}	0.33	O_{169}	0.28	O_{182}	0.29
	O_{206}	0.28				
μ_2-O atoms in $\{Mo_4O_6\}$ units	O_{19}	0.42	O_{32}	0.46	O_{52}	0.41
	O_{129}	0.46	O_{134}	0.40		

TGA curves

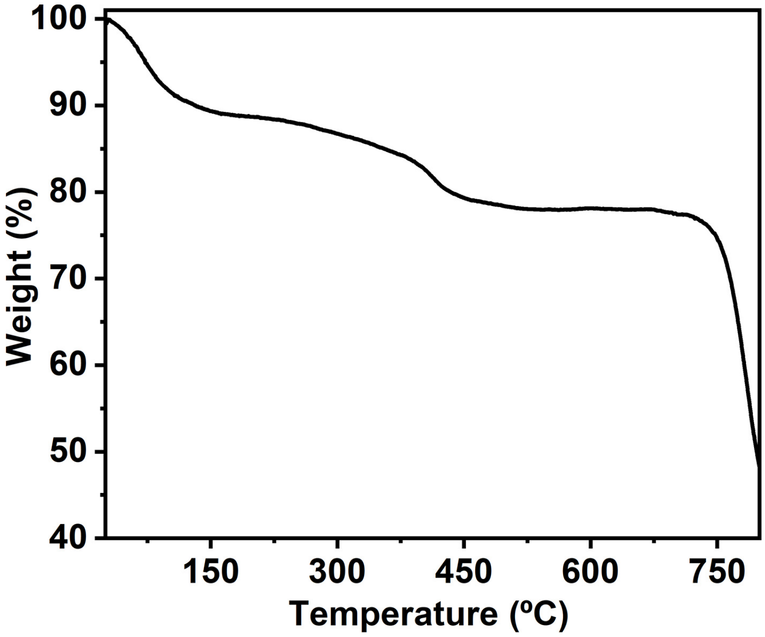

Supplementary Figure 3. TGA curve. TGA curve for **1**. 9.38% weight loss corresponds to ~122 H_2O.

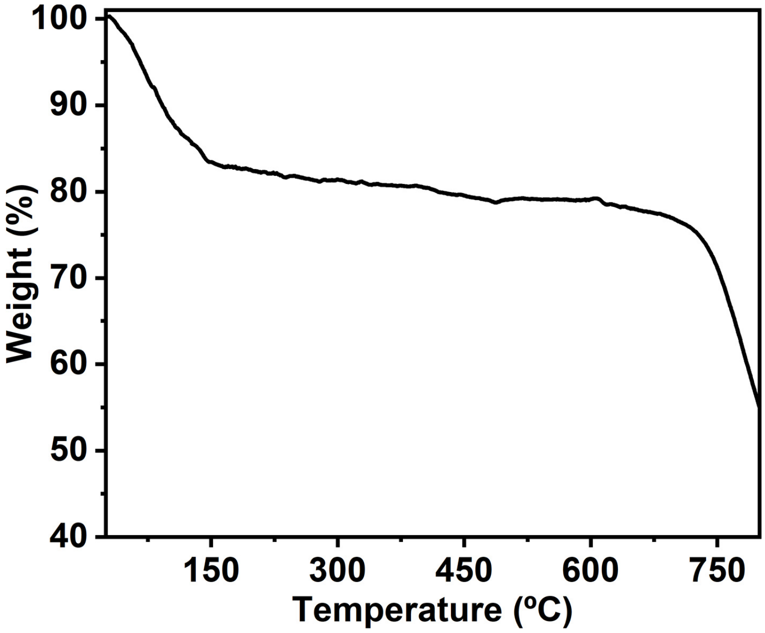

Supplementary Figure 4. TGA curve. TGA curve for **2**. 14.5% weight loss corresponds to ~170 H_2O.

Summary of structure analysis of **1** and **2**

{Mo_{132}}: The formula of **1** is calculated based upon the well-established wheel- and ball-shaped polyoxomolybdates. Its overall reduction state (60 electrons reduced) was confirmed using above techniques. This cluster should be divided into two parts during the structure analysis, the {Mo_{110}} framework featuring an unprecedented archetypal decamer and two {Mo_{11}} caps on both sides of {Mo_{110}}. As for the {Mo_{110}} wheel, the Mo^V centers (totally 50 Mo^V) are located at (1) {Mo_5O_6}-type double cubanes as expected (Supplementary Table 2, 20 electrons reduced), (2) the edge-shared {Mo_2} units (Supplementary Table 3, 20 electrons reduced) and (3) {Mo_1} centers (Supplementary Table 4, 10 electrons reduced). As for the {Mo_{11}} caps, each contains 5 Mo^V centers according to the BVS calculation (Supplementary Table 5). Thus it can be seen that there are totally 60 Mo^V centers in **1** theoretically, which is in line with the experimental results with 60 electrons reduced. A careful analysis of the bond length of Mo-O bonds and bond valence sums reveals 10 singly and 12 doubly protonated oxygen atoms (coordination water). Singly protonated are the 10 equivalent μ_3-O atoms situated in the incomplete double-cubane-type {Mo_5O_6} (Supplementary Table 2). All the other bridging O atoms are considered as O^{2-} except for the 10 equivalent μ_3-O atoms. Several terminal O atoms in two {Mo_{11}} caps that have bond lengths of Mo-O in the range of 2.0 to 2.2 Å could be assigned as coordination water (Supplementary Table 6). Additionally, 10 SO_4^{2-} with half site-occupancy disorder and 20 CH_3COO^- are attached to the internal and external surface of capped wheel **1**, respectively. In this way, we could determine the overall charge of polyoxoanion **1a** as -52.

About 44 water molecules and one Na^+ cation were found from the Fourier maps, however, there are still a very large accessible solvent voids in the crystal structure caculated by SQUEEZE subroutine of PLATON software, indicating that some more solvent molecules and cations should exist in the structure, but cannot be found from the weak residual electron peaks. Based on the TGA curve and elemental analyses, another 21 Na^+ cation and 78 water molecules were included into the molecular formula directly. To balance the negative charge of each cluster, 30 H^+ are proposed as counterions based on the elemental analysis result of H. The count electrons for Na^+ cation and water molecule are both 10. The aforementioned total count electrons are *ca.* 990. The void contains appropriative electronic density (*ca.* 990) to accommodate all cations/water molecules that cannot be determined crystallographically. The free water molecules in this formula were further confirmed by TGA curve, a total weight loss of 9.38 %, which corresponds to ~122 guest water molecules. Elemental analysis result of Mo confirms the cluster consists of 132 Mo atoms, consistent with single-crystal x-ray diffraction.

The archetypal ring {Mo_{110}} in **1** consists of 10 sets of three different building block types: {Mo_8}, {Mo_1} and {Mo_2}. We can determine the composition of {Mo_{110}} as {$Mo^{VI/V}{}_8O_{26}(\mu_3O)_2H(S_{0.5}O_3)(CH_3COO)_2Mo^{VI/V}$}$_{10}${$Mo^V{}_2O_4$}$_{10}$. The formula of {$Mo_{11}$} cap could be evaluated as {$Mo^VO^\#$}$_5${(Mo^{VI})$Mo^{VI}{}_5O_{21}(H_2O)_6$} based on the aggregation of one {Mo_6} units and five {$Mo_1^\#$} units featuring edge-shared coordination modes with {Mo_1} units in {Mo_8} building blocks of {Mo_{110}} wheel. Taking into consideration of the obtained information from the above calculations along with Single-crystal x-ray diffraction, elemental analyses, bond valence sum analysis, UV-vis, TGA, redox titration and SQUEEZE calculation, it is possible to determine the overall building-block scheme and overall formula for **1** as

$Na_{22}H_{30}\{Mo_1\}_{10}\{Mo_8\}_{10}\{Mo_2\}_{10}\{Mo_1^\#\}_{10}\{Mo_6\}_2\cdot 122H_2O \equiv$

$Na_{22}H_{30}\{Mo^{VI/V}{}_8O_{26}(\mu_3O)_2H(S_{0.5}O_3)(CH_3COO)_2Mo^{VI/V}\}_{10}\{Mo^V{}_2O_4\}_{10}\{Mo^VO^\#\}_{10}\{(Mo^{VI})Mo^{VI}{}_5O_{21}(H_2O)_6\}_2\cdot 122H_2O$.

$\{Ce_{11}Mo_{96}\}$: The formula of half-closed **2** is calculated based upon its parent **1**. This cluster should also be divided into two parts during the structure analysis, the $\{Mo_{85}\}$ framework and one $\{Mo_{11}\}$ cap on one side of $\{Mo_{85}\}$. As for the $\{Mo_{85}\}$ wheel, the Mo^{V} centers (totally 25 Mo^{V}) are located at (1) $\{Mo_4O_6\}$/$\{Mo_5O_6\}$-type double cubanes (Supplementary Table 2, 20 electrons reduced) and (2) $\{Mo_1\}$ centers (Supplementary Table 4, 5 electrons reduced). As for the $\{Mo_{11}\}$ cap, it contains 5 Mo^{V} centers according to the BVS calculation (Supplementary Table 5). Thus it can be seen that there are totally 30 Mo^{V} centers in $\{Mo_{85}\}$. A careful analysis of the bond length of Mo-O bonds and bond valence sums reveals 46 doubly protonated oxygen atoms (coordination water). No singly protonated μ_3-O atoms situated in the incomplete double-cubane-type $\{Mo_5O_6\}$ could be identified (Supplementary Table 2). In addition, the determination of doubly protonated oxygen atoms should be the following two cases: (1) Several terminal O atoms in $\{Mo_8\}$ of $\{Mo_{85}\}$ wheel and $\{Mo_{11}\}$ cap that have bond lengths of Mo-O in the range of 2.0 to 2.2 Å could be assigned as coordination water (Supplementary Table 6 and Table 7, totally 36 doubly protonated oxygen atoms). (2) The μ_2-O atoms in $\{Mo_4O_6\}$ of $\{Mo_{85}\}$ wheel could be assigned as coordination water (Supplementary Table 7, totally 10 doubly protonated oxygen atoms, BVS less than 0.50 were assigned to H_2O). Such μ_2-O atom is not unique because it has already been considered as coordination water molecules in classical Mo-S system (*Chem. Soc. Rev.* **41**, 7335–7353(2012)). All the other bridging O atoms are considered as O^{2-}. There are 11 Ce atoms with 59 coordination water molecules. Additionally, 8 SO_4^{2-} are attached to the internal surface of half-closed **2**. In this way, we could determine the overall charge of polyoxoanion **2a** as -9.

About 27 water molecules and one Na^+ cation were found from the Fourier maps, however, there are still a very large accessible solvent voids in the crystal structure as presented in **1**, indicating that some more solvent molecules and cations should exist in the structure, but cannot be found from the weak residual electron peaks. Based on the TGA curve and elemental analyses, another 2 Na^+ cation and 143 water molecules were included into the molecular formula directly. To balance the negative charge of each cluster, 6 H^+ are proposed as counterions based on the elemental analysis result of H. The free water molecules in this formula were further confirmed by TGA curve, a total weight loss of 14.5 %, which corresponds to ~170 guest water molecules. Elemental analysis result of Mo confirms the cluster consists of 96 Mo atoms and 11 Ce atoms, consistent with single-crystal x-ray diffraction.

The $\{Mo_{85}\}$ wheel consists of two different building block types: 10 $\{Mo_8\}$ and 5 $\{Mo_1\}$. The $\{Mo_1\}$ units in five $\{Mo_5O_6\}$-type double cubanes are missing compared to its parent wheel in **1**. We can determine the composition of $\{Mo_{85}\}$ as $\{Mo^{VI/V}{}_8O_{24}(H_2O)_5\}_5\{Mo^{VI/V}{}_8O_{26}(\mu_3O)_2(H_2O)_3Mo^{VI/V}\}_5$. The formula of $\{Mo_{11}\}$ cap could be evaluated as $\{(Mo^{VI})Mo^{VI}{}_5O_{21}(H_2O)_6\}\{(SO_4)Mo^{V}O^{\#}\}_5$ based on the aggregation of one $\{Mo_6\}$ units and five $\{Mo_1^{\#}\}$ units featuring edge-shared coordination modes with $\{Mo_1\}$ units in $\{Mo_8\}$ building blocks of $\{Mo_{85}\}$ wheel. The 11 Ce atoms with 59 coordination water molecules as well as 3 SO_4^{2-} could be described as $\{Ce_8(H_2O)_{46}\}\{Ce(H_2O)_3\{SO_4\}\}_3$. Taking into consideration of the obtained information from the above calculations along with Single-crystal x-ray diffraction, elemental analyses, bond valence sum analysis, Uv-vis, TGA and redox titration, it is possible to determine the overall building-block scheme and overall formula for $\{Ce_{11}Mo_{96}\}$ as $Na_3H_6\{Mo_8\}_{10}\{Mo_1\}_5\{Mo_6\}\{Mo_1^{\#}\}_5\{Ce\}_{11}\cdot 170H_2O$ ≡ $Na_3H_6\{Mo^{VI/V}{}_8O_{24}(H_2O)_5\}_5\{Mo^{VI/V}{}_8O_{26}(\mu_3O)_2(H_2O)_3Mo^{VI/V}\}_5\{(Mo^{VI})Mo^{VI}{}_5O_{21}(H_2O)_6\}\{(SO_4)Mo^{V}O^{\#}\}_5\{Ce_8(H_2O)_{46}\}\{Ce(H_2O)_3\{SO_4\}\}_3\cdot 170$ H_2O.

Single-crystal X-ray diffraction: Single-crystal X-ray diffraction data for **1** and **2** were recorded on a Bruker Apex CCD II area-detector diffractometer with graphite-monochromated $Mo_{K\alpha}$ radiation (λ = 0.71073 Å) at 173(2) K. Absorption corrections were applied using multi-scan technique and performed by using the SADABS program (Sheldrick, G. *SADABS*; ver. 2.10; University of Gottingen: Göttingen, Germany, 2003). The structures were solved by direct methods and refined on F^2 by full-matrix least squares methods by using the SHELXTL (A.L. Spek, *Acta Cryst.* C71, 9–18(2015)) minimization on Olex 2 software package (O.V. Dolomanov, L.J. Bourhis, R. J. Gildea, J. A. K. Howard, H. Puschmann, *J. Appl. Crystallogr. 42*, 339–341(2009)).

Supplementary Table 8. Crystallographic data for **1** and **2**.

CCDC	2225462 (**1**)	2225463 (**2**)
Empirical formula	$C_{40}H_{368}Mo_{132}Na_{22}O_{576}S_5$	$Ce_{11}Mo_{96}Na_3O_{589}S_8H_{548}$
Formula weight	23397.48	21053.36
Temperature (K)	173.0 K	173.0 K
Crystal system	Tetragonal	Orthorhombic
Space group	$P4_2/ncm$	*Pnma*
a/Å	51.4842(6)	40.0797(12)
b/Å	51.4842(6)	41.9928(12)
c/Å	22.4533(6)	34.5552(10)
α(°)	90	90
β(°)	90	90
γ(°)	90	90
V/Å^3	59515(2)	58158(3)
ρ_{calc}(g/cm^3)	2.611	2.248
μ/mm^{-1}	23.363	24.252
F(000)	44328.0	40346.0
θ (°)	5.428 to 127.618	3.978 to 101.276
Reflections collected	263269	201733
Independent reflections	24951 [R_{int} = 0.0672]	31105 [R_{int} = 0.0880]
GOF	1.075	1.040
Final R indices [I>2sigma(I)]	R_1 = 0.0763, wR_2 = 0.1692	R_1 = 0.0875, wR_2 = 0.2411
R indices (all data)	R_1 = 0.0815, wR_2 = 0.1720	R_1 = 0.1076, wR_2 = 0.2625

$^aR_1 = \Sigma||F_o| - |F_c||/\Sigma|F_o|$, $^bwR_2 = [\Sigma w(F_o^2 - F_c^2)^2/\Sigma w(F_o^2)^2]^{1/2}$.

Supplementary Figures

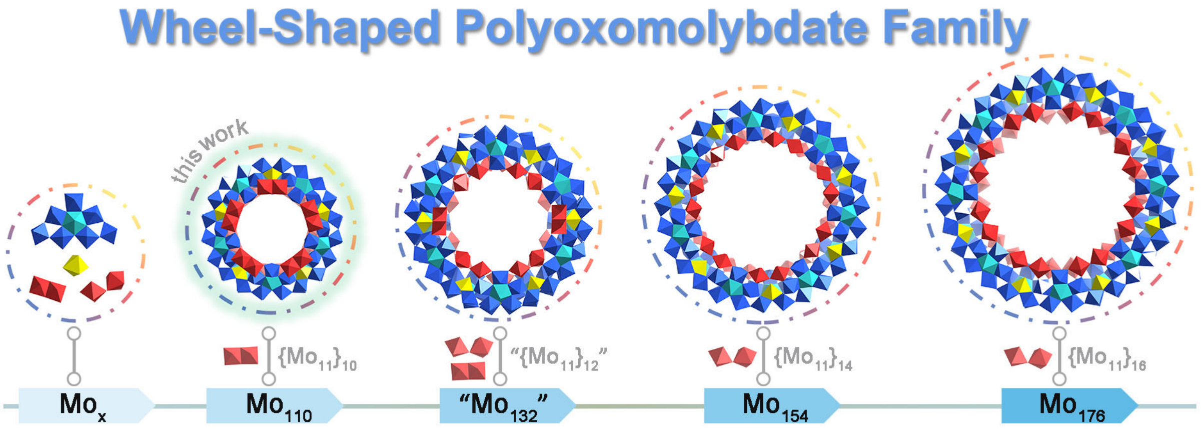

Supplementary Figure 5. Wheel-shaped polyoxomolybdate family. The Mo wheel family based on different $\{Mo_2\}$ units and the fundamental $\{Mo_{11}\}$ building blocks. The $\{MoO_7\}$ are shown in the cyan polyhedra and the $\{MoO_6\}$ are shown in the blue/red/yellow polyhedra.

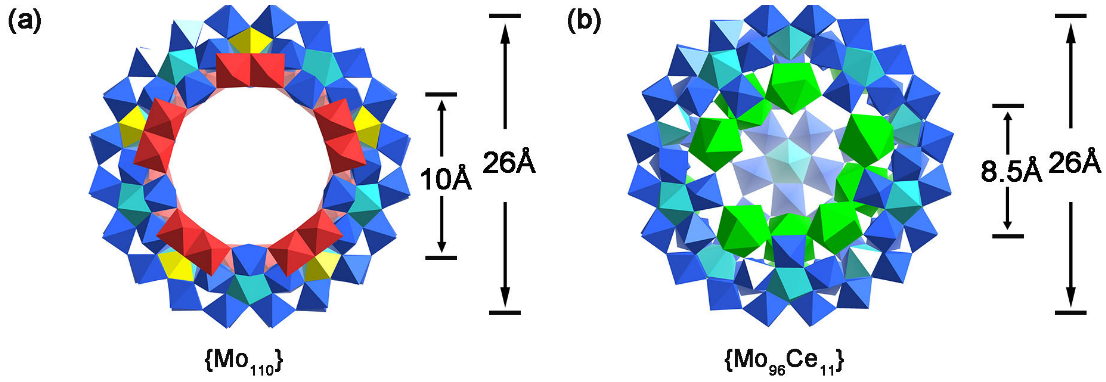

Supplementary Figure 6. The outer and inner ring diameter. (a) Polyhedral representations of outer (~26Å) and inner (~10Å) ring diameter in $\{Mo_{110}\}$ of **1a**. (b) Polyhedral representations of outer (~26Å) and inner (~8.5Å) ring diameter in $\{Mo_{96}Ce_{11}\}$ of **2a**. Color code: Mo blue/red/cyan/yellow, Ce green.

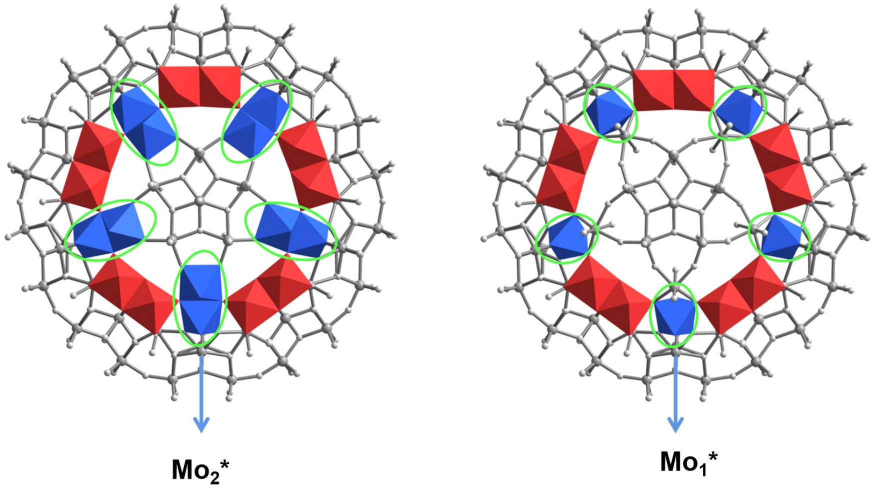

Supplementary Figure 7. The binding modes of simple Mo species. The binding modes for $\{Mo_1^*\}$ and $\{Mo_2^*\}$ in **1a**. The $\{Mo_1^*\}$ and $\{Mo_2^*\}$ are shown in the blue polyhedra.

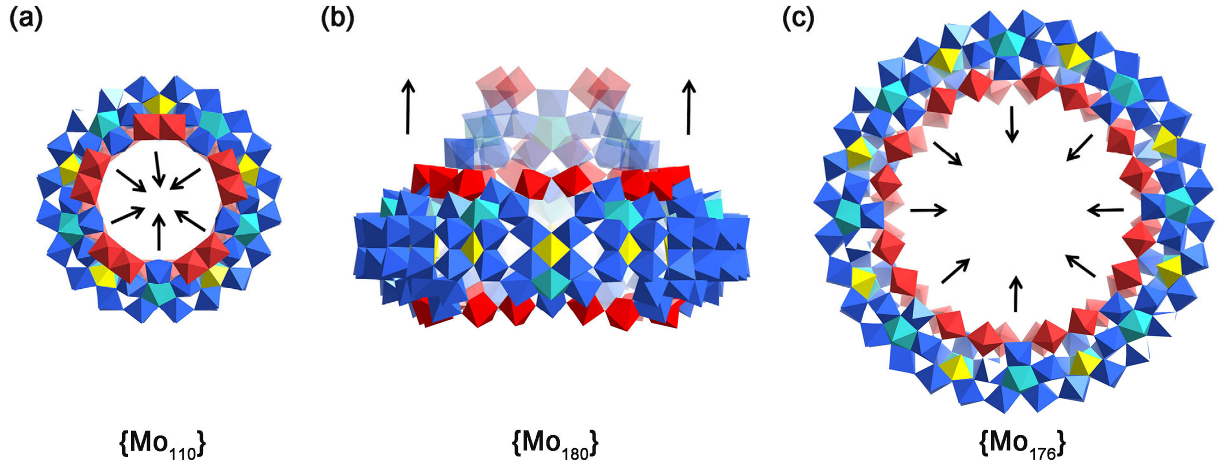

Supplementary Figure 8. The in-plane growth pattern in Mo wheels. The {Mo_1}-driven in-plane growth in {Mo_{110}} of **1** (a), {Mo_{180}} (b) and the {Mo_2}-mediated in-plane growth in {Mo_{248}} (c). The {MoO_7} are shown in the cyan polyhedra and The {MoO_6} are shown in the blue/red/yellow polyhedra.

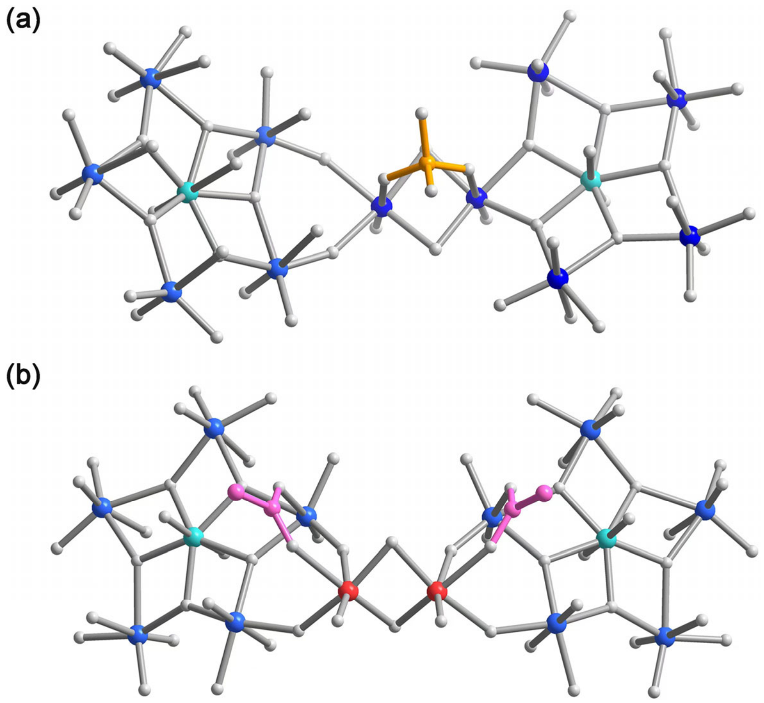

Supplementary Figure 9. The binding modes of {SO_4^{2-}} (a) and {CH_3COO^-} (b) ligands in 1a. The {CH_3COO^-} groups are shown in the pink ball-and-stick representation and the {SO_4^{2-}} is shown in the orange ball-and-stick representation. Color code: Mo blue/cyan, O grey/pink.

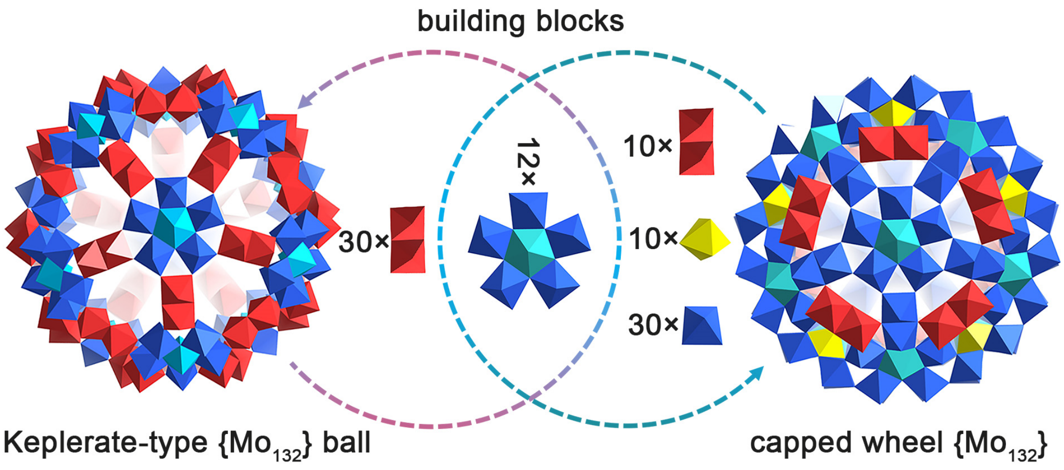

Supplementary Figure 10. Mo_{132} ball *vs* Mo_{132} capped wheel. Polyhedral representations of Mo_{132} ball *vs* Mo_{132} capped wheel and their Mo-based building blocks. Color code: Mo blue/red/cyan/yellow.

The definition of Mo_{132} ball *vs* Mo_{132} capped wheel quasi-isomers.

In chemistry, isomers are molecules with identical formulas but distinct structures. The subject of isomerism has been well established in atomically precise nanoclusters, mainly focused on Au, Ag and Cu nanoclusters, see *J. Am. Chem. Soc.* **138**, 1482−1485(2016), *J. Am. Chem. Soc.* **140**, 42, 13590–13593(2018), *Chem. Sci.* **10**, 8685−8693(2019), *etc*. These Au, Ag and Cu clusters structural isomers are those clusters that follow the same compositions but adopt different configurations. In the strictest point of view, all elements including metals and ligands in these nanocluster structural isomers should be identical. However, if the isomers share the same number of metal centers with different geometrical structures and capping ligands, they could be considered as "quasi-isomers", see *Chem. Mater.* **33**, 39–62(2021). Molecular isomerism in polyoxometalates is rather rare, see *Chem. Eur. J.* **19**, 2976–2981(2013), *Angew. Chem. Int. Ed.* **57**, 2972–2975(2018), in this regard, according to the definition of isomers and quasi-isomers in atomically precise nanoclusters, we concluded that "polyoxometalate quasi-isomers" may share same number of metal centers with similar metal-oxygen bonding modes (*e.g.* the same valence state of metal centers and characteristic building blocks) by ignoring different outer ligands. In this work, 132-Mo-atom clusters possess identical reduction degree (45%, $Mo^{V}_{60}Mo^{VI}_{72}$) and characteristic {Mo(Mo)$_5$} motifs with similar connection rules but having different geometrical structures (*e.g.* ball *vs.* capped wheel), thus we think that they are 132-Mo-atom "quasi-isomers". This pair of 132-Mo-atom "quasi-isomers" constitutes quasi-isomerism in polyoxometalates.

The comparison of the synthetic conditions for Mo_{132} ball *vs* Mo_{132} capped wheel.

The Na-salt of {Mo_{132}} ball was obtain by the reaction of $Na_2MoO_4{\cdot}2H_2O$ (0.628 mmol), $N_2H_6SO_4$ (0.123 mmol) and CH_3COONa (3.243 mmol) in 5 ml water at room temperature, see *J. Am. Chem. Soc.* **138**, 10623−10629(2016), while the Na-salt of {Mo_{132}} capped wheel was synthesized from a one-pot reaction of an acidified aqueous mixture of $Na_2MoO_4{\cdot}2H_2O$ (0.72 mmol), $Na_2S_2O_4$ (0.60 mmol) and $CH_3COONa{\cdot}3H_2O$ (0.70 mmol) upon heating. The synthetic conditions of the isomers remain many commonalities, such as the molybdenum raw material, acidic environment, the existence of reducing agent, and the assistance of small organic ligands (*e.g.* CH_3COO^-), *etc*. However, the differences for the synthetic conditions are much worth discussing,

which had been summarized as the following four tips: (a) different reaction temperatures. {Mo_{132}} ball was synthesized at room temperature (without heating) and {Mo_{132}} capped wheel could only be obtained under hydrothermal conditions. This is in line with the fact that hydrothermal reactions may bring novel building blocks or special structure of molybdenum oxide clusters (*e.g.* capped wheel configuration). (b) different pH value. Wheel-shaped molybdenum oxide clusters are commonly synthesized at a specific low pH value (< 2.5), see *Chem. Soc. Rev.* **41**, 7431-7463(2012), and {Mo_{132}} ball was obtained at pH ~4.00, pH value is a known crucial parameter in polyoxomolybdate chemistry as a series of available Mo-based building units toward the formation of the final structures are driven by it. (c) different reducing agent. A series of hydrazide-containing compounds had been used in the construction of high-nuclearity molybdenum oxide clusters, including {Mo_{132}} ball. Sodium hydrosulfite had been employed as effective reducing agent in the construction of high-nuclearity molybdenum oxide clusters, as our previous work, see *Chem. Sci.* **13**, 4573-4580(2022). (d) different concentrations of Mo-containing solution. The concentration of Mo-containing solution for {Mo_{132}} ball is a little high than that for {Mo_{132}} capped wheel. Higher concentrations were found to promote crystal growth quickly, while lower concentrations lead to good crystals but with a longer crystallization time and lower yields, see *J. Am. Chem. Soc.* **141**, 1242−1250(2019).

Theoretical analysis of Mo_{132} ball *vs* Mo_{132} capped wheel

The analysis of electronic distribution properties for this pair of 132-Mo-atom POMs by theoretical data may give rise to valuable information to clarify "their difference in delocalization of the reducing electrons". We choose {$H_{42}Mo_{16}O_{67}$} units (Supplementary Figure 11a) in {Mo_{132}} ball for theoretical analysis, and in {Mo_{132}} capped wheel, we choose both {$H_{11}Mo_5O_{20}$} units (Supplementary Figure 12a) in wheel and {$H_{62}Mo_{26}O_{107}$} units (Supplementary Figure 13a) that contains reduced {Mo^V_2} units for theoretical analysis (the reasons are shown below), respectively. Detailed theoretical analysis had been presented as follows:

(a) {Mo_{132}} ball. This structure possesses a metal-oxo framework with high crystal symmetry that contains 132 Mo atoms consisting of twelve {Mo_6} pentagonal units and thirty {Mo_2} linkers. As previous report, the reducing electrons in {Mo_{132}} ball are localized in reduced and isolated {Mo^V_2} units, see *Bull. Jpn. Soc. Coord. Chem.* **78**, 11-17(2021), *Angew. Chem. Int. Ed.* **42**, 2085–2090(2003). So we selected the classical {$H_{42}Mo_{16}O_{67}$} unit (Supplementary Figure 11a) involving five {Mo^V_2} and one central {(Mo)Mo_5} unit in {Mo_{132}} ball as reasonable models for theoretical analysis, in which the unsaturated oxygen is terminated with H. The single-point calculations were carried out at the (U)B3LYP/6-31G(d)(H, O)/Lanl2DZ(Mo) level. Supplementary Figure 11c shows the occupied molecular orbitals of the classical {$H_{42}Mo_{16}O_{67}$} unit in {Mo_{132}} ball, obvious $Mo^V\cdots Mo^V$ interactions could be identified. The electron density map of {$H_{42}Mo_{16}O_{67}$} unit in {Mo_{132}} ball (Supplementary Figure 11b) highlights that the distribution of electron density is mainly focus on {Mo^V_2} units as expected. Based on current theoretical analysis, it is not difficult to find that the reducing electrons are localized in reduced and isolated {Mo^V_2} units in {Mo_{132}} ball.

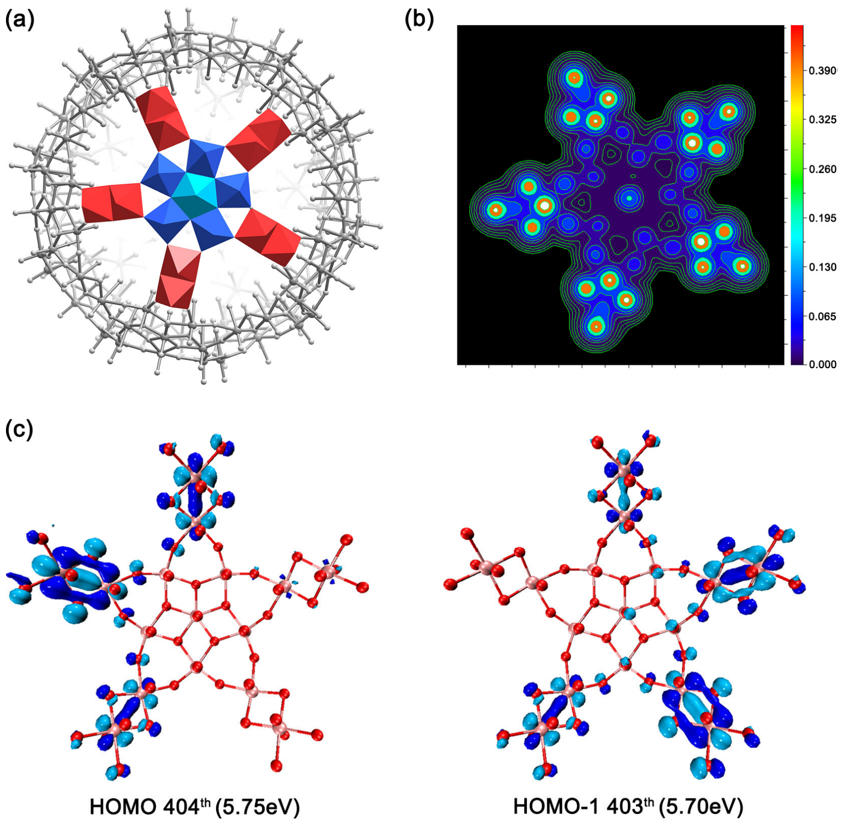

Supplementary Figure 11. Theoretical analysis of {Mo_{132}} ball. (a) The calculation model {$H_{42}Mo_{16}O_{67}$} unit taken from {Mo_{132}} ball. (b) The electron density map of {$H_{42}Mo_{16}O_{67}$} unit. (c) Occupied molecular orbitals of {$H_{42}Mo_{16}O_{67}$} unit. The orbital energy is presented in parenthesis.

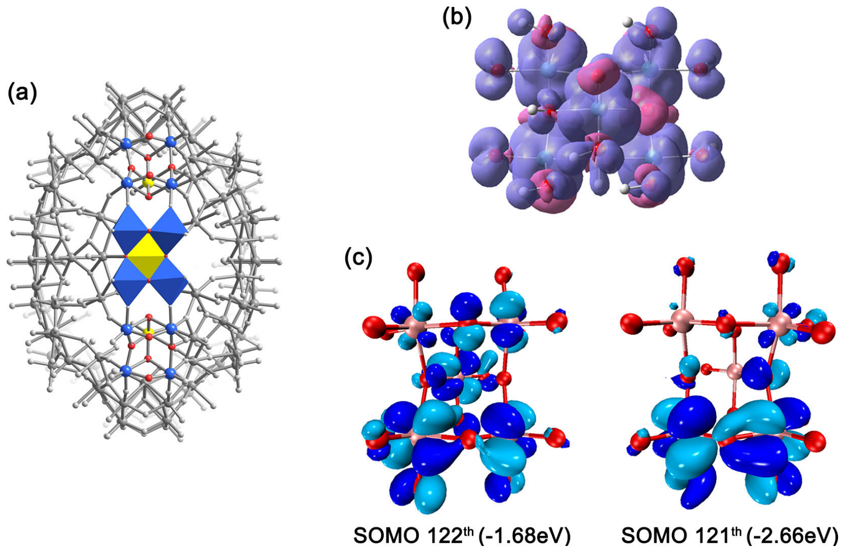

Supplementary Figure 12. Theoretical analysis of {Mo_{132}} capped wheel. (a) The calculation model {$H_{11}Mo_5O_{20}$} unit taken from {Mo_{132}} capped wheel. (b) Spin density and (c) SOMOs of {$H_{11}Mo_5O_{20}$}. The orbital energy is presented in parenthesis.

(b) {Mo_{132}} capped wheel. This structure reported here possesses a capped wheel configuration, which consists of a Mo wheel and two Mo caps. The Mo^V centers in both wheel and caps should be fully considered during the analysis of the delocalization of electrons. In fact, the number and position of reduced Mo^V centers of both compounds had already been well-presented in the part of formula determination. Based on this, we selected the {$H_{11}Mo_5O_{20}$} unit (Supplementary Figure 12a) in wheel and {$H_{62}Mo_{26}O_{107}$} (Supplementary Figure 13a) that contains reduced {Mo^V_2} units as ideal models for theoretical analysis, in which the unsaturated oxygen is terminated with H. The single-point calculations were carried out at the (U)B3LYP/6-31G(d)(H, O)/Lanl2DZ(Mo) level. As for the {$H_{11}Mo_5O_{20}$} unit in Mo wheel part, previous reports show that most of Mo wheel are blue colored as the electrons from reduction are delocalized over the wheel structure, in the central belt spanning the ring, namely {Mo_5O_6}-type double cubanes, which is confirmed by bond valence sum calculations, redox titration as well as UV/Vis spectroscopy, see *Acc. Chem. Res.* **33**, 2-10(2000), *J. Am. Chem. Soc.* **142**, 13982−13988(2020). As shown in Supplementary Figure 12b, spin density of {$H_{11}Mo_5O_{20}$} shows two single electrons distribute on five Mo atoms. Also, two single occupied molecular orbitals (SOMOs) of {$H_{11}Mo_5O_{20}$} are delocalized at five Mo atoms (Supplementary Figure 12c), consistent with the above-discussion. As for the {$H_{62}Mo_{26}O_{107}$} unit (Supplementary Figure 13a), which contains ten sets of {Mo^V_2} units, the reduced electrons are expected to be localized in {Mo^V_2} units. The occupied molecular orbitals are located at {Mo^V_2} units, indicating obvious $Mo^V \cdots Mo^V$ interactions (Supplementary Figure 13c). Furthermore, the electron density map clearly shows that the electron density distributes on all {Mo^V_2} units (Supplementary Figure 13b). Based on current theoretical analysis for both rational wheel and cap species in {Mo_{132}} capped wheel, it is not difficult to find that the reducing electrons are not only localized in reduced and isolated {Mo^V_2} units as well as delocalized over all {Mo_5O_6}-type double cubanes in the ring.

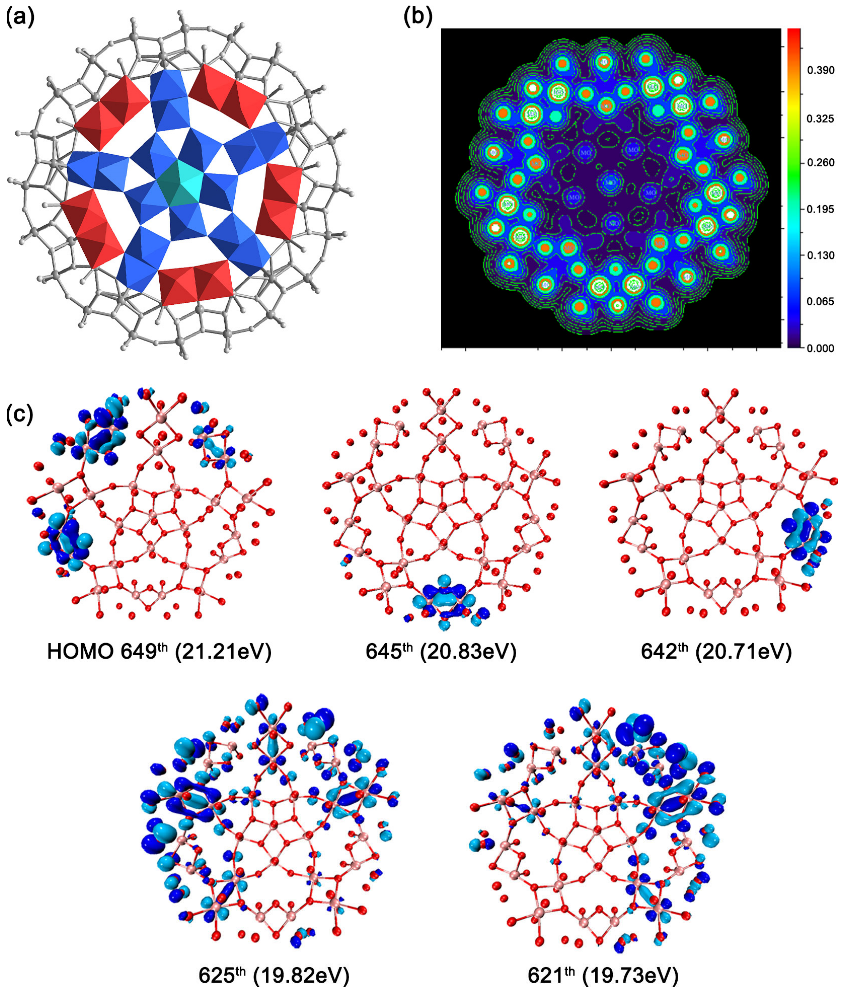

Supplementary Figure 13. Theoretical analysis of {Mo_{132}} capped wheel. (a) The calculation model {$H_{62}Mo_{26}O_{107}$} unit taken from {Mo_{132}} capped wheel. (b) The electron density map of {$H_{62}Mo_{26}O_{107}$} unit. (c) Occupied molecular orbitals of {$H_{62}Mo_{26}O_{107}$} unit. The orbital energy is presented in parenthesis.

In summary, to a certain degree, the current theoretical data support the fact that Mo_{132} ball and the Mo_{132} capped wheel show different delocalization of the reducing electrons. If the reducing electrons are simply delocalized over the ring, it exhibits classical molybdenum blue, while if the reducing electrons are localized in reduced {Mo^{V}_{2}} units at different positions, the molybdenum cluster exhibits molybdenum red or molybdenum brown, see *Angew. Chem. Int. Ed.* **61**, e202201672(2022). Thus it can be seen that when electrons are both delocalized over the wheel and reduced {Mo^{V}_{2}} units, this leads to the unprecedented new ‘Mo Green’ reported in current work. From this pair of 132-Mo-atom cluster, we also found that the color displayed by reduced giant polyoxomolybdates could not only be related to the degree of Mo reduction but also the factor of the location of reduction.

Photochemical performance of Mo_{132} isomerism

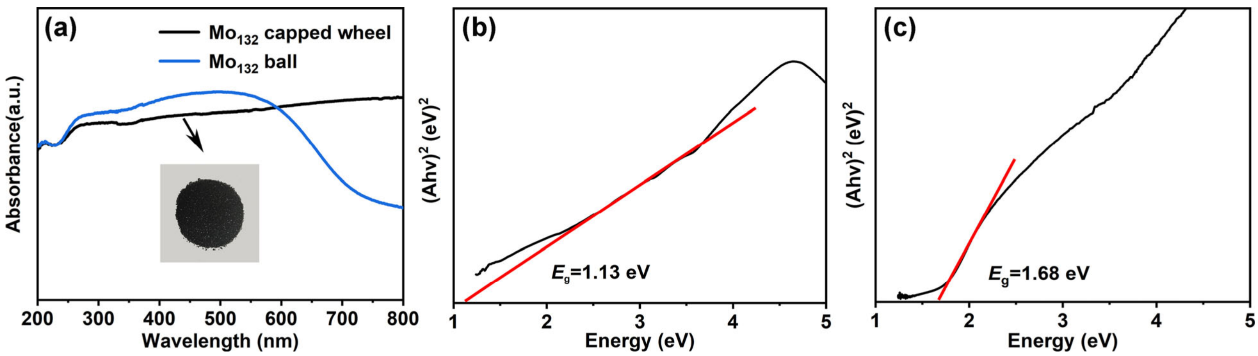

Supplementary Figure 14. UV/Vis absorption spectra in the solid state. The diffuse reflectance spectra (a) and corresponding well-defined optical band-gap energies of **1** (b) and Keplerate-type $\{Mo_{132}\}$ ball (c).

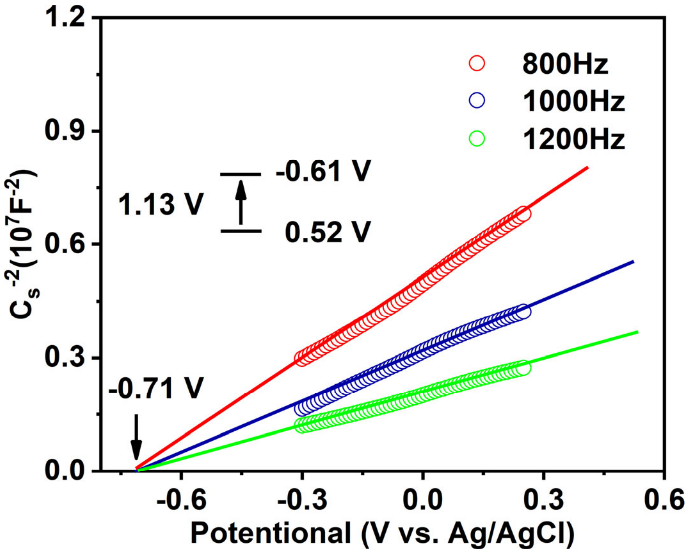

Supplementary Figure 15. Mott–Schottky plots for 1.

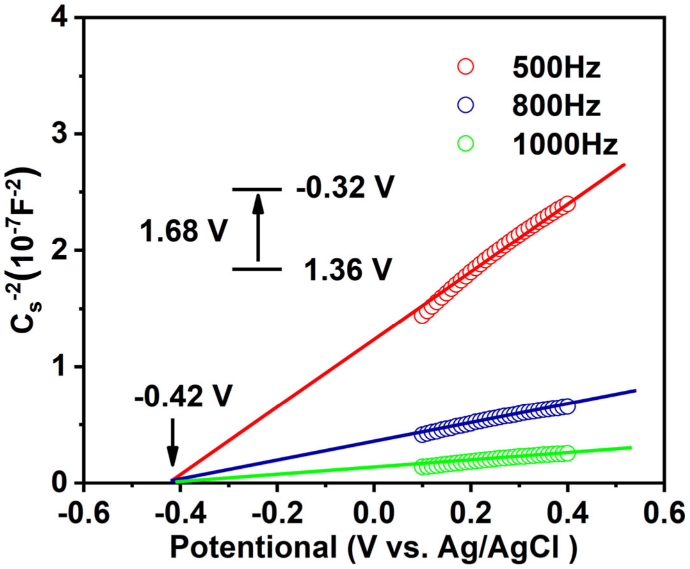

Supplementary Figure 16. Mott–Schottky plots for Keplerate-type $\{Mo_{132}\}$ ball.

Stability of **1** in solution

Preparation of tetrabutylammonium (TBA) salt of **1** (TBA-**1**). **1** (3 mg) was immersed and slowly dissolved in 10 mL of deionized water overnight to form dark green aqueous solution. After that, this dark green aqueous solution was added to an aqueous solution of tetrabutylammonium bromide (200 mg in 5 mL water), and dark green precipitation was formed immediately. The precipitation of TBA-**1** was collected by centrifugation, and washed with H_2O and EtOH.

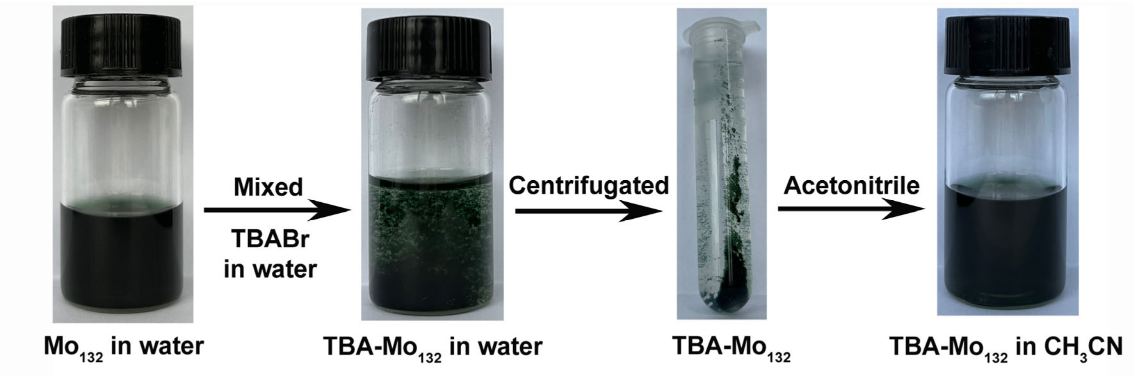

Supplementary Figure 17. Preparation of TBA salt of 1. The procedures of the preparetion of TBA-**1**.

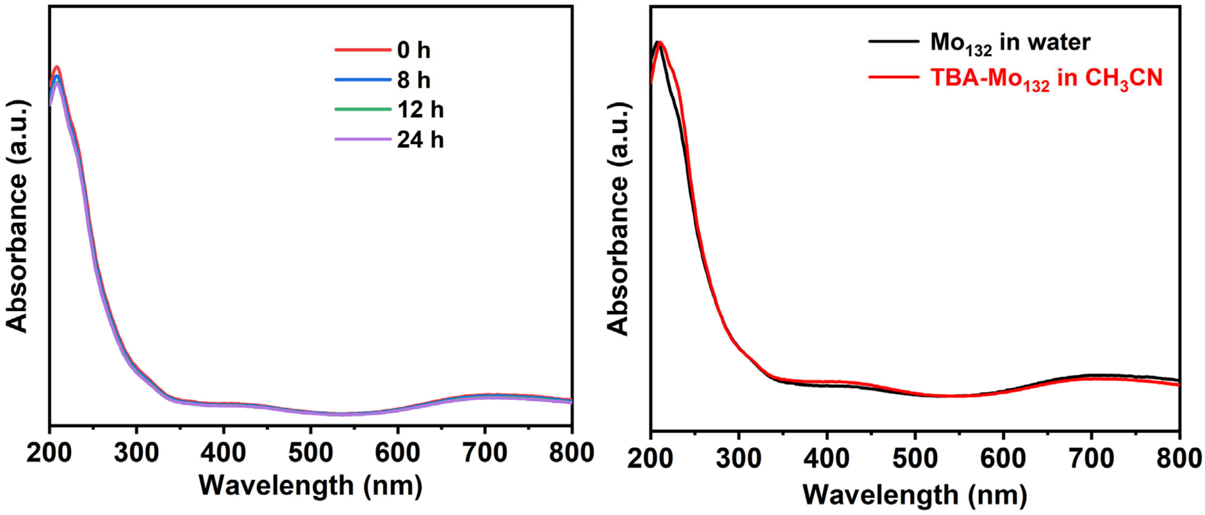

Supplementary Figure 18. UV/Vis absorption spectra in solution. (Left) Monitoring of the UV-Vis absorption of **1** in aqueous solution for 24h. (Right) UV−vis absorption spectra of **1** in H_2O and TBA-**1** in acetonitrile.

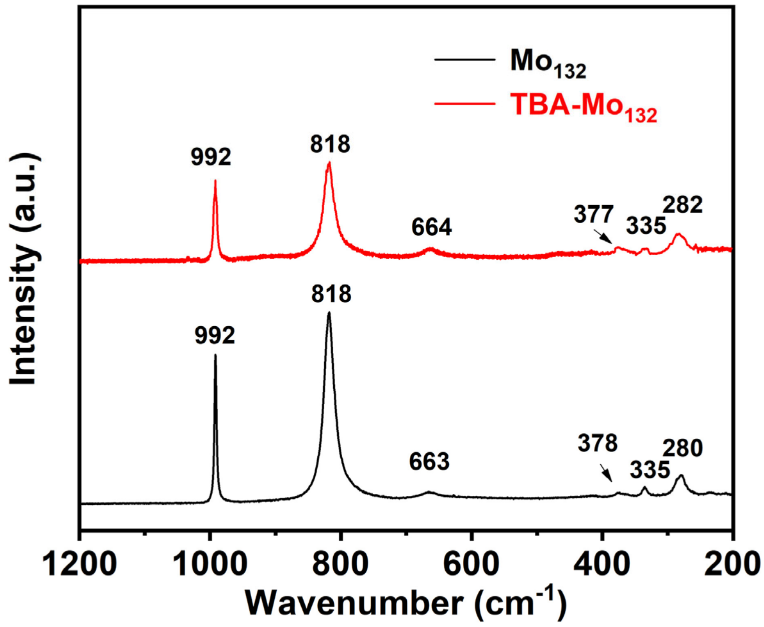

Supplementary Figure 19. Raman spectra. Raman spectra of the crystal samples of **1** and TBA-**1**.

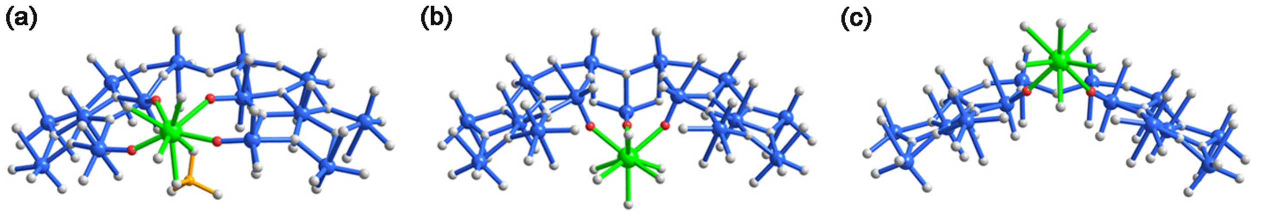

Supplementary Figure 20. The binding modes of Ce centers and {SO_4^{2-}} ligand. The ball-and-stick representations of 4-, 3- and 2-connected rods in Ce centers with 'On' (a), 'Inner' (b) and 'Outer' (c) binding modes, respectively. The binding modes for {μ_2-η^1:η^1:η^0:η^0-SO_4^{2-}} ligand (a) in **2a**.

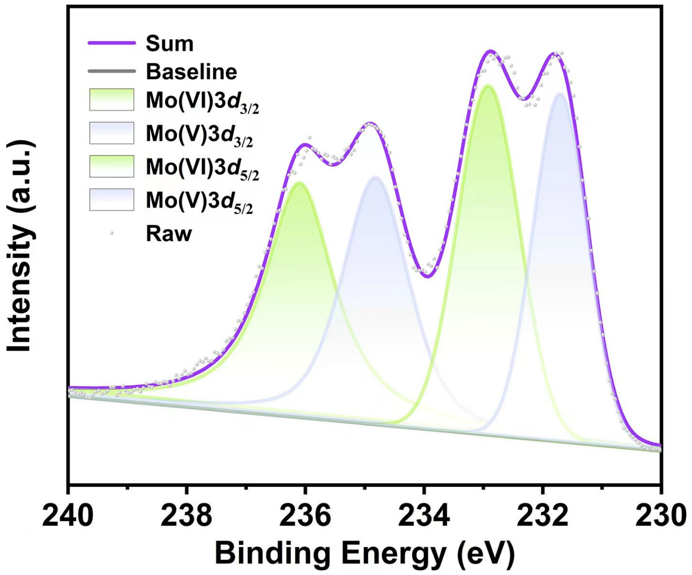

Supplementary Figure 21. XPS spectrum of Mo in 1. Four peaks at 236.1, 234.9, 232.9, and 231.7 eV, which correspond to the binding energies of Mo(VI)-3$d_{3/2}$, Mo(V)-3$d_{3/2}$, Mo(VI)-3$d_{5/2}$, and Mo(V)-3$d_{5/2}$, respectively, thus demonstrating that the valence states of Mo atoms in **1** are + V and + VI.

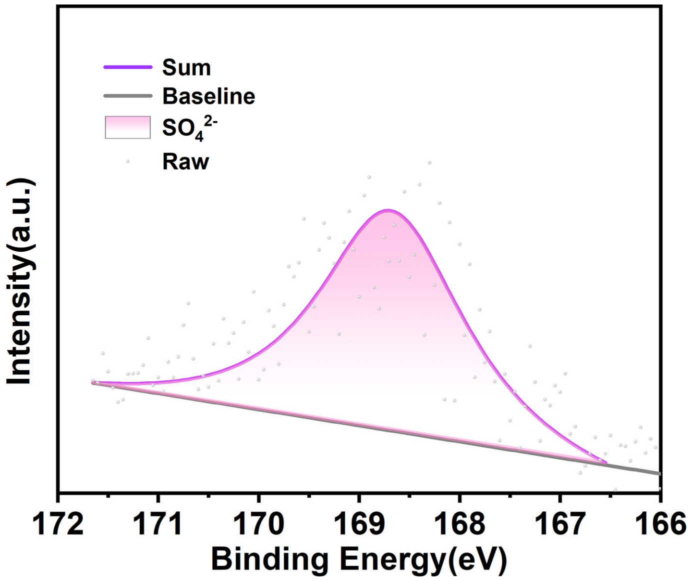

Supplementary Figure 22. XPS spectrum of S in 1. The S 2*p* spectrum displays one peak at 168.7 corresponded to the S 2*p* binding energies of S^{6+} for SO_4^{2-} in **1**.

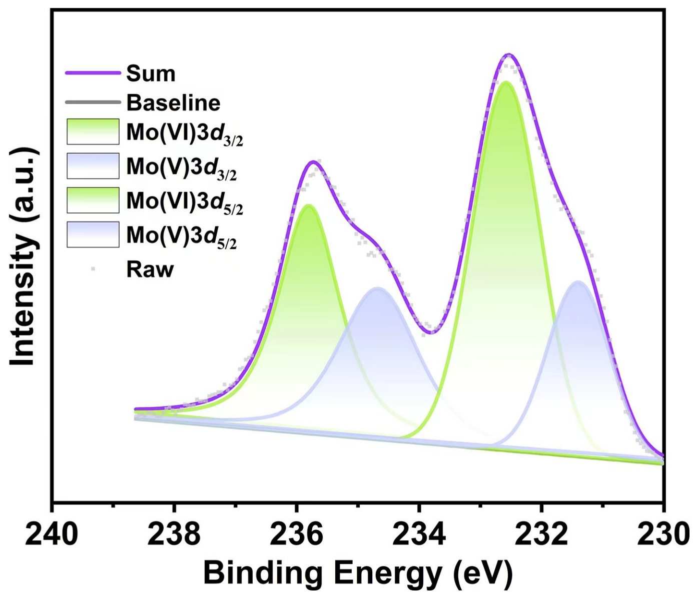

Supplementary Figure 23. XPS spectrum of Mo in 2. Four peaks at 235.8, 234.7, 232.6, and 231.4 eV, which correspond to the binding energies of Mo(VI)-3$d_{3/2}$, Mo(V)-3$d_{3/2}$, Mo(VI)-3$d_{5/2}$, and Mo(V)-3$d_{5/2}$, respectively, thus demonstrating that the valence states of Mo atoms in **2** are + V and + VI.

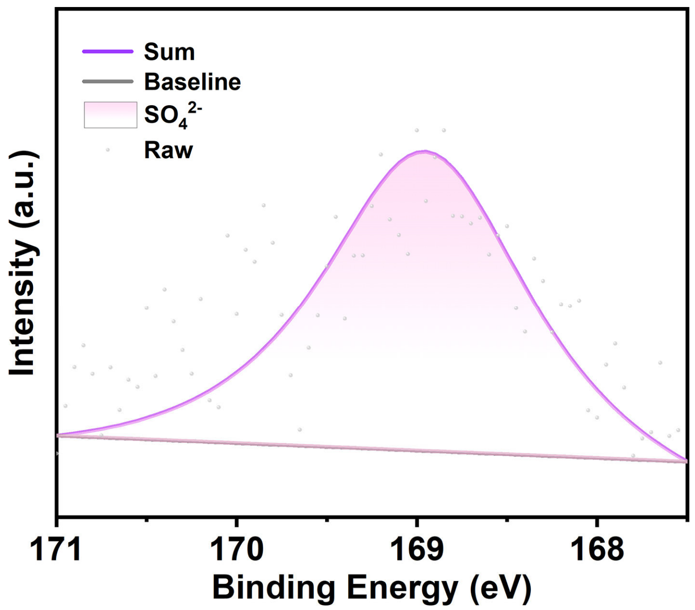

Supplementary Figure 24. XPS spectrum of S in 2. The S 2*p* spectrum displays one peak at 169.0 corresponded to the S 2*p* binding energies of S^{6+} for SO_4^{2-} in **2**.

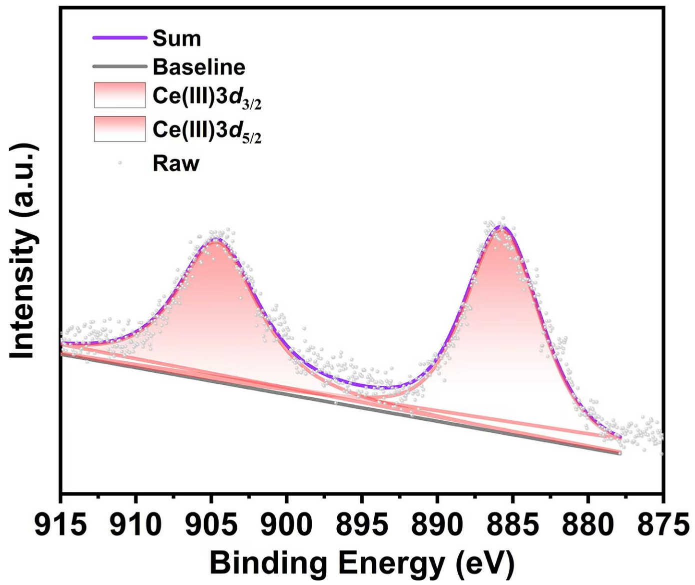

Supplementary Figure 25. XPS spectrum of Ce in 2. The peaks around 904.7 and 885.8 eV in the energy regions of Ce 3$d_{3/2}$ and Ce 3$d_{5/2}$ correspond to Ce^{3+}. It is noting that the Ce 3*d* XPS spectra of Ce-containing compounds are known to be complicated due to the hybridization of the Ce 4*f* with ligand orbitals and fractional occupancy of the 4*f* valence orbitals, see *Langmuir* **12**, 1794-1799(1996); *J. Phys. Chem. C* **117**, 9520−9528(2013); *Inorg. Chem.* **53**, 9486−9497(2014).

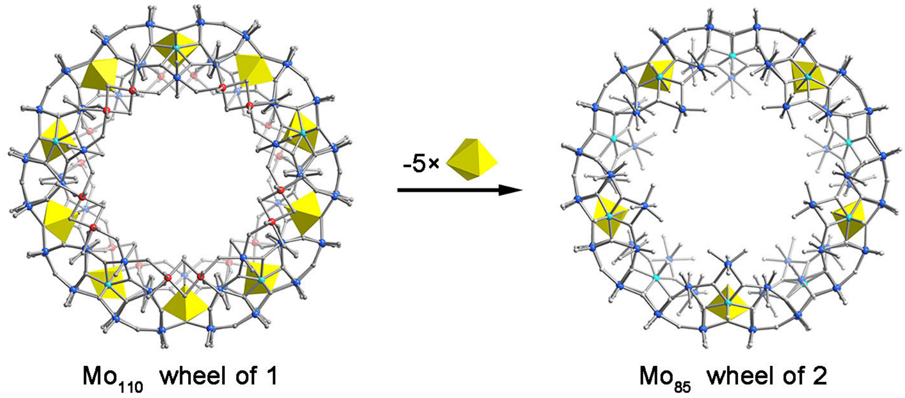

Supplementary Figure 26. Structural evolution from Mo_{110} wheel in 1 to Mo_{85} wheel in 2. The missing of five Mo atoms of the wheel in **1a** to access the main wheel of **2a** without altering the main Mo framework. The above Mo atoms are shown in the yellow polyhedra.

Supplementary Table 9. Bond lengths of Ce centers (Å) in **2a**.

Bond	Length (Å)	Bond	Length (Å)	Bond	Length (Å)
Ce(1)-O(51)	2.544(11)	Ce(1)-O(81)	2.474(10)	Ce(1)-O(103)	2.555(11)
Ce(1)-O(151)	2.476(11)	Ce(1)-O(171)	2.527(10)	Ce(1)-O(207)	2.583(11)
Ce(1)-O(209)	2.564(12)	Ce(1)-O(211)	2.547(12)	Ce(1)-O(246)	2.508(10)
Ce(3)-O(99)	2.450(15)	Ce(3)-O(223)	2.50(2)	Ce(3)-O(221)	2.58(2)
Ce(3)-O(108)	2.577(10)	Ce(3)-O(161)	2.657(12)	Ce(3)-O(181)	2.546(14)
Ce(3)-O(199)	2.68(2)	Ce(3)-O(200)	2.660(10)	Ce(4)-O(244$^{\#1}$)	2.458(10)
Ce(4)-O(244)	2.458(10)	Ce(4)-O(66)	2.572(13)	Ce(4)-O(101)	2.543(16)
Ce(4)-O(185)	2.541(13)	Ce(4)-O(185$^{\#1}$)	2.541(13)	Ce(4)-O(188)	2.582(10)
Ce(4)-O(188$^{\#1}$)	2.582(10)	Ce(4)-O(201)	2.560(15)	Ce(5)-O(195)	2.661(15)
Ce(5)-O(195$^{\#1}$)	2.661(15)	Ce(5)-O(45)	2.487(19)	Ce(5)-O(104$^{\#1}$)	2.494(12)
Ce(5)-O(104)	2.494(12)	Ce(5)-O(179)	2.429(13)	Ce(5)-O(179$^{\#1}$)	2.429(13)
Ce(5)-O(191)	2.61(2)	Ce(5)-O(193)	2.50(2)	Ce(6)-O(217)	2.415(16)
Ce(6)-O(236)	2.328(13)	Ce(6)-O(49)	2.618(18)	Ce(6)-O(85)	2.537(13)
Ce(6)-O(87)	2.673(14)	Ce(6)-O(97)	2.443(16)	Ce(6)-O(109)	2.568(17)
Ce(6)-O(183)	2.513(15)	Ce(6)-O(203)	2.620(14)	Ce(8)-O(215)	2.601(13)
Ce(8)-O(13)	2.564(13)	Ce(8)-O(27)	2.632(16)	Ce(8)-O(53)	2.560(12)
Ce(8)-O(93)	2.469(13)	Ce(8)-O(163)	2.504(10)	Ce(8)-O(173)	2.480(10)
Ce(8)-O(187)	2.549(14)	Ce(8)-O(213)	2.515(12)	Ce(9)-O(219)	2.575(18)
Ce(9)-O(55)	2.508(18)	Ce(9)-O(77)	2.522(11)	Ce(9)-O(79)	2.525(11)
Ce(9)-O(105)	2.575(13)	Ce(9)-O(107)	2.585(14)	Ce(9)-O(111)	2.611(19)
Ce(9)-O(189)	2.530(16)	Ce(9)-O(205)	2.621(13)		

Symmetry code： $^{\#1}$+X,1/2-Y,+Z.

Supplementary Table 10. Bond lengths of S centers (Å) in **2a**.

Bond	Length (Å)	Bond	Length (Å)	Bond	Length (Å)
S(1)-O(84)	1.524(10)	S(1)-O(92)	1.456(10)	S(1)-O(135)	1.438(11)
S(1)-O(244)	1.461(11)	S(2)-O(49)	1.613(18)	S(2)-O(95)	1.514(15)
S(2)-O(97)	1.493(15)	S(2)-O(197)	1.580(19)	S(3)-O(3$^{\#1}$)	1.495(14)
S(3)-O(3)	1.495(14)	S(3)-O(191)	1.51(2)	S(3)-O(193)	1.49(2)
S(4)-O(35)	1.450(15)	S(4)-O(181)	1.448(13)	S(4)-O(181$^{\#1}$)	1.448(13)
S(4)-O(232)	1.499(15)	S(5)-O(43)	1.467(12)	S(5)-O(47)	1.458(15)
S(5)-O(99)	1.520(15)	S(5)-O(242)	1.479(12)		

Symmetry code： $^{\#1}$+X,1/2-Y,+Z.

Proton conductivity measurement

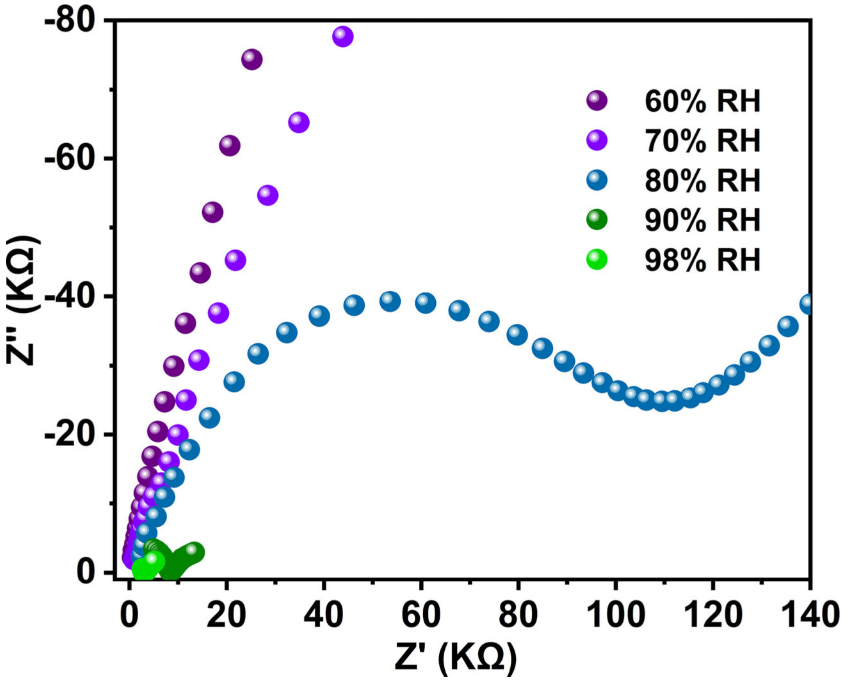

Supplementary Figure 27. Proton conductivity of 1 at various RH. The Nyquist plots of **1** at 30°C and various RH (60-98%).

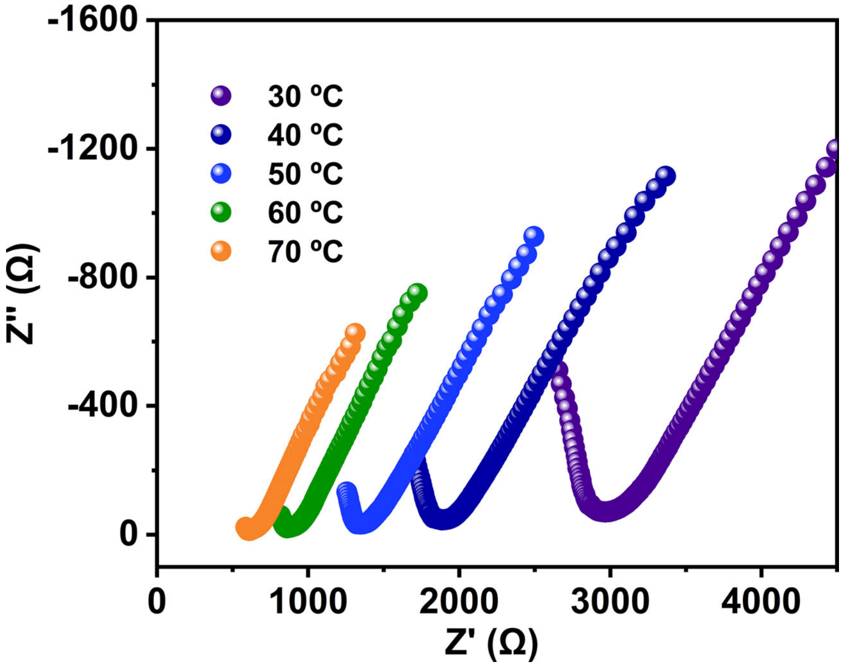

Supplementary Figure 28. Proton conductivity of 1 at various temperatures. The Nyquist plots of **1** at 98% RH and various temperatures (30-70°C).

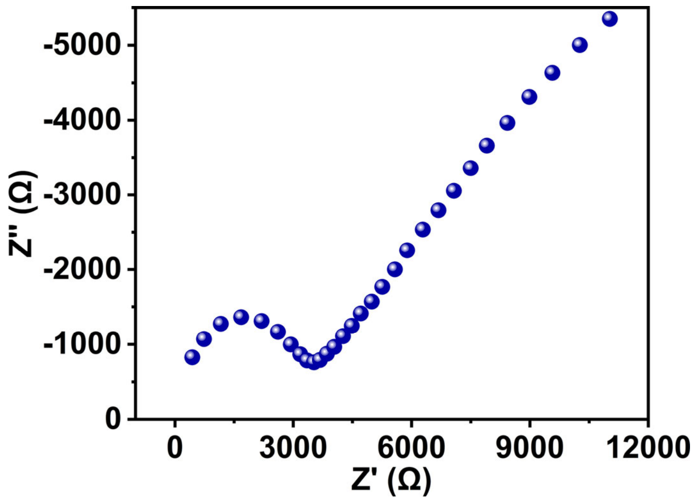

Supplementary Figure 29. Nyquist plots for 2 at 80% RH in D_2O. 2 exhibited a proton conductivity of 2.02×10^{-4} $S\cdot cm^{-1}$ at 80% RH (D_2O, 30 °C, Supplementary Figure 29). The conductivity value was lower than that of measured in H_2O under same condition. The decreased proton conductivity in deuterate water was caused by the isotopic effect of deuterium. The isotopic effect supported our hypothesis that ionic conductivity was caused by proton transfer. See *Nat. Mater.* **15**, 722–726(2016), *Chem. Sci.* **10**, 556–563(2019), *Inorg. Chem. Front.* **5**, 1213-1217(2018), *etc.*

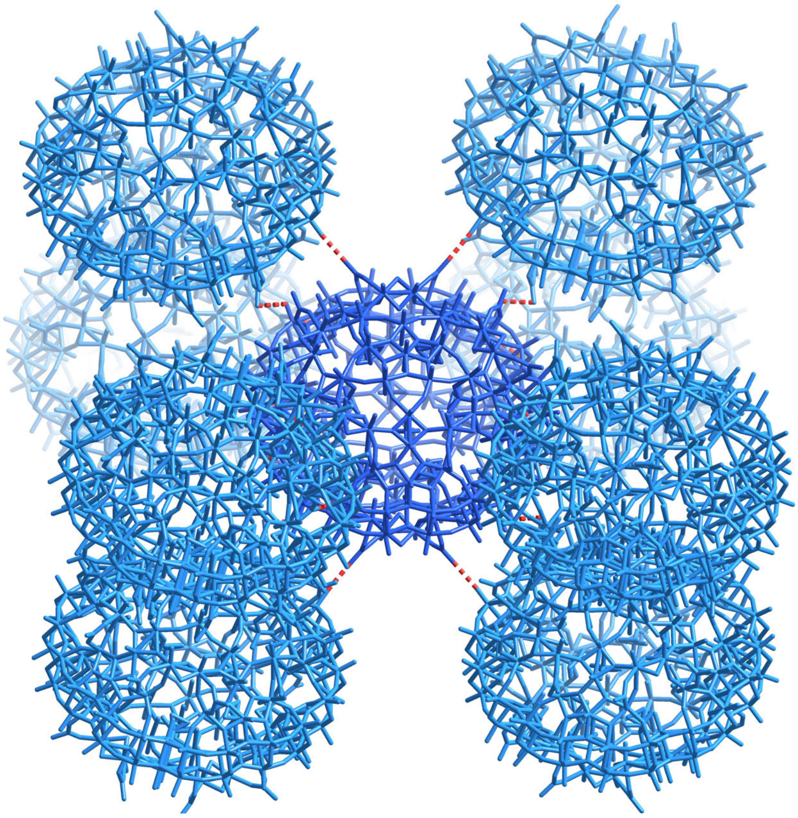

Supplementary Figure 30. View of packing diagram of 3D structure in 1a. Relative positions of eight adjacent Mo_{132} units (light blue) centered around one Mo_{132} (dark blue), the hydrogen bond distances are shown in red.

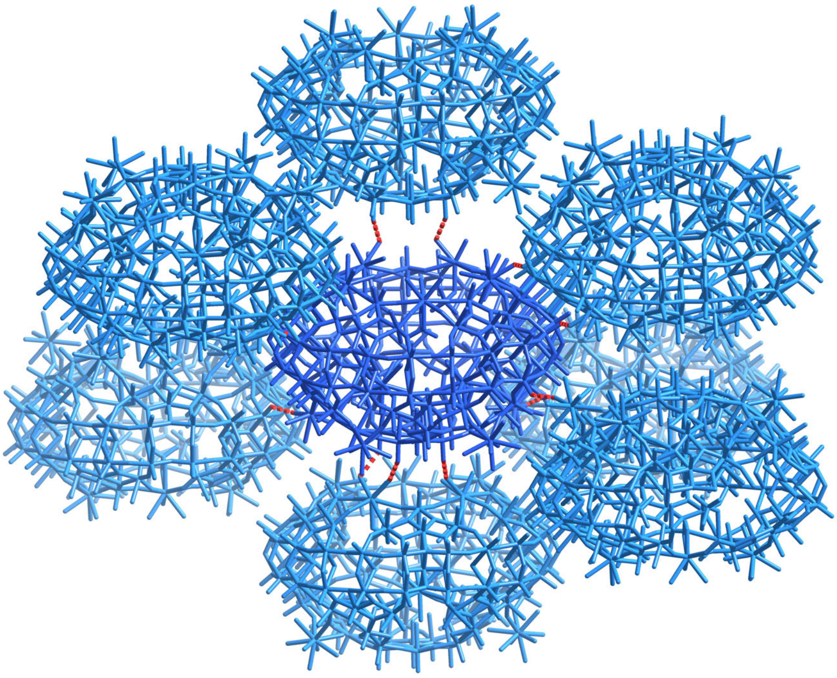

Supplementary Figure 31. View of packing diagram of 3D structure in 2a. Relative positions of seven adjacent $Ce_{11}Mo_{96}$ units (light blue) centered around one $Ce_{11}Mo_{96}$ (dark blue), the hydrogen bond distances are shown in red.

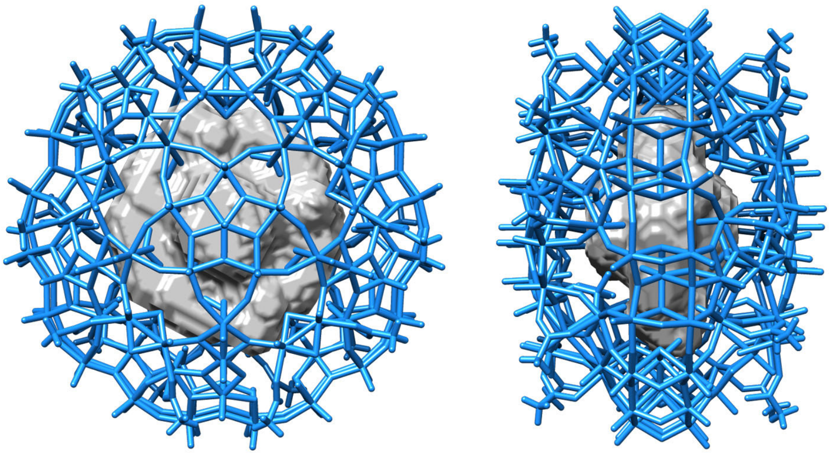

Supplementary Figure 32. The void space of 1a. The void space (grey cavity) of **1a** calculated by VOIDOO based upon its X-ray crystal structure. See *Science* **366**, 613–620(2019); *J. Am. Chem. Soc.* **142**, 18060–18072(2020). All atoms are shown in light blue.

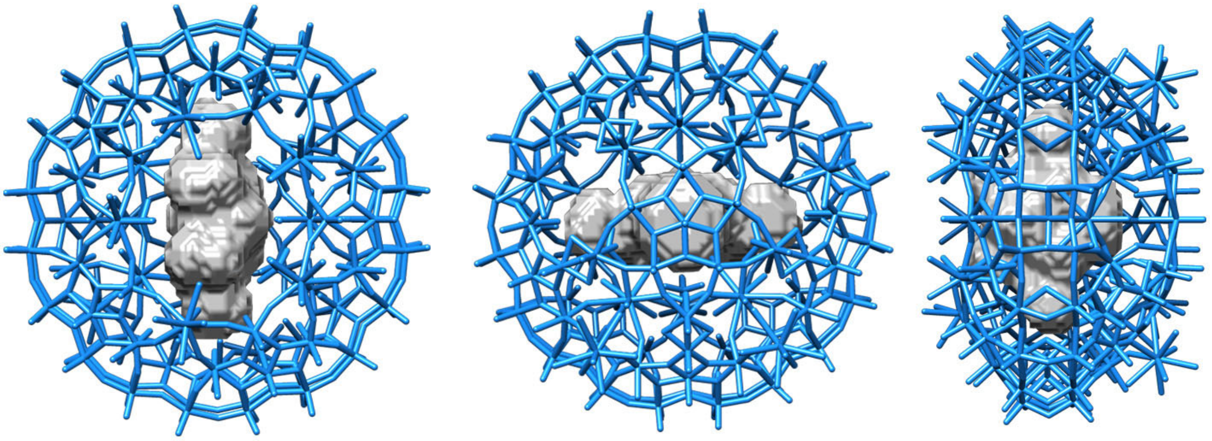

Supplementary Figure 33. The void space of 2a. The void space (grey cavity) of **2a** calculated by VOIDOO based upon its X-ray crystal structure. See *Science* **366**, 613–620(2019); *J. Am. Chem. Soc.* **142**, 18060–18072(2020). All atoms are shown in light blue.

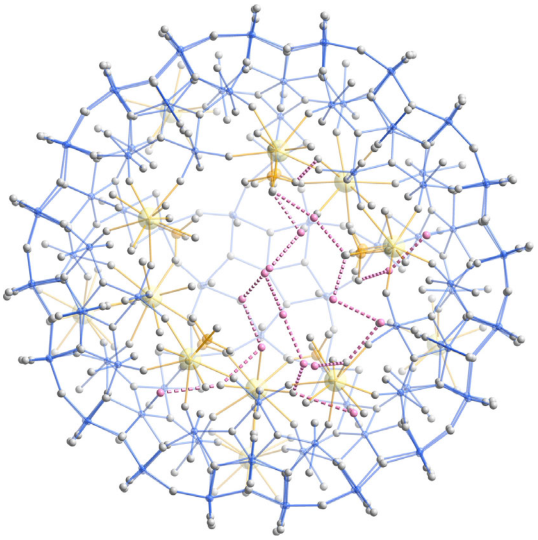

Supplementary Figure 34. The hydrogen bonding networks inside the cavity. The short O···O distances with free water molecules inside the cavity of **2a**. Color code: Mo blue, S orange, O gray, Ce yellow. Free water molecules are shown in pink.

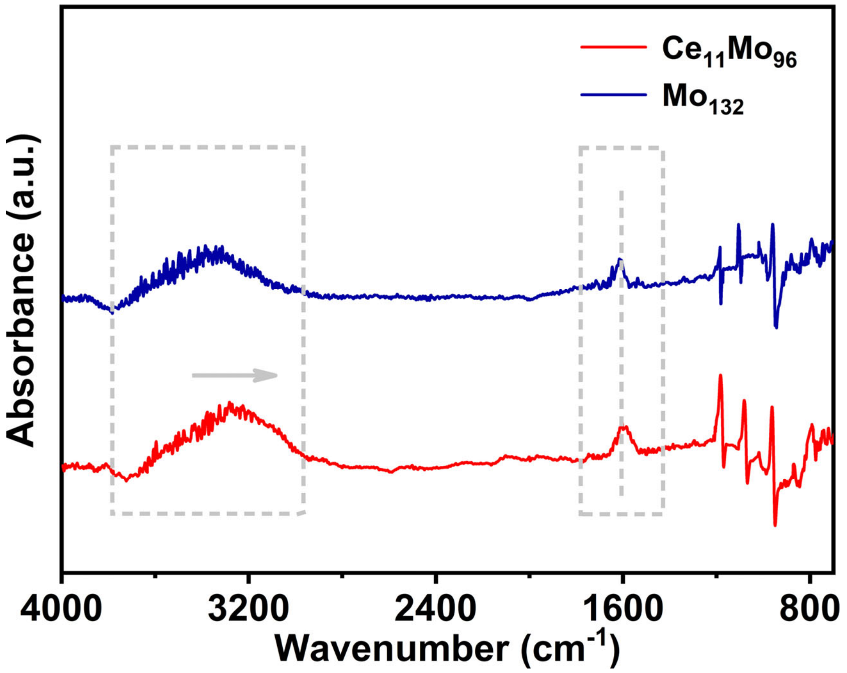

Supplementary Figure 35. In situ IR spectra. In situ IR spectra of **1** (blue) and **2** (red) under a water vapor pressure of 2.5 kPa.

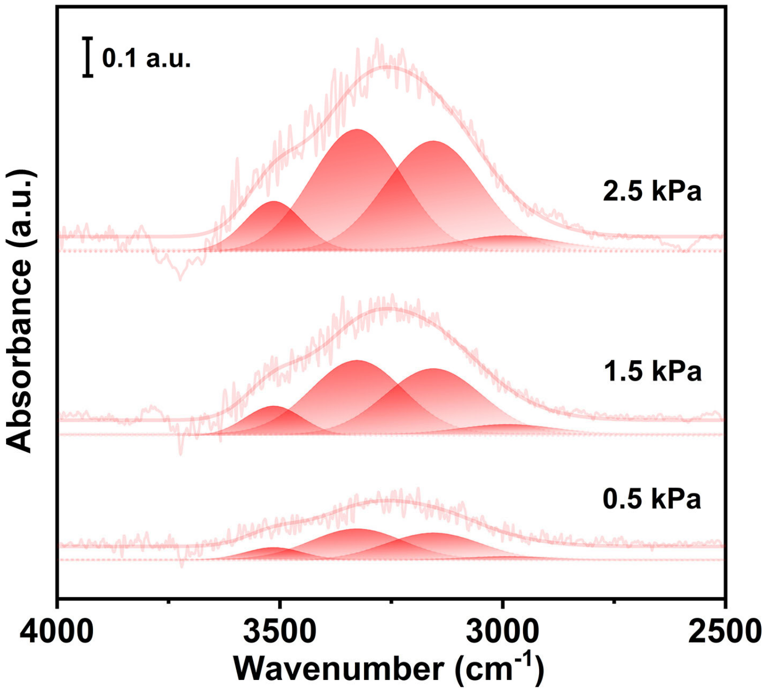

Supplementary Figure 36. In situ IR spectra of 2 under different water vapor pressure. In situ IR spectra of **2** under a water vapor pressure of 0.5 kPa, 1.5 kPa, and 2.5 kPa in the OH stretching region. The observed bands were well reproduced by the sum (solid lines) of four Gaussian peaks (broken lines).

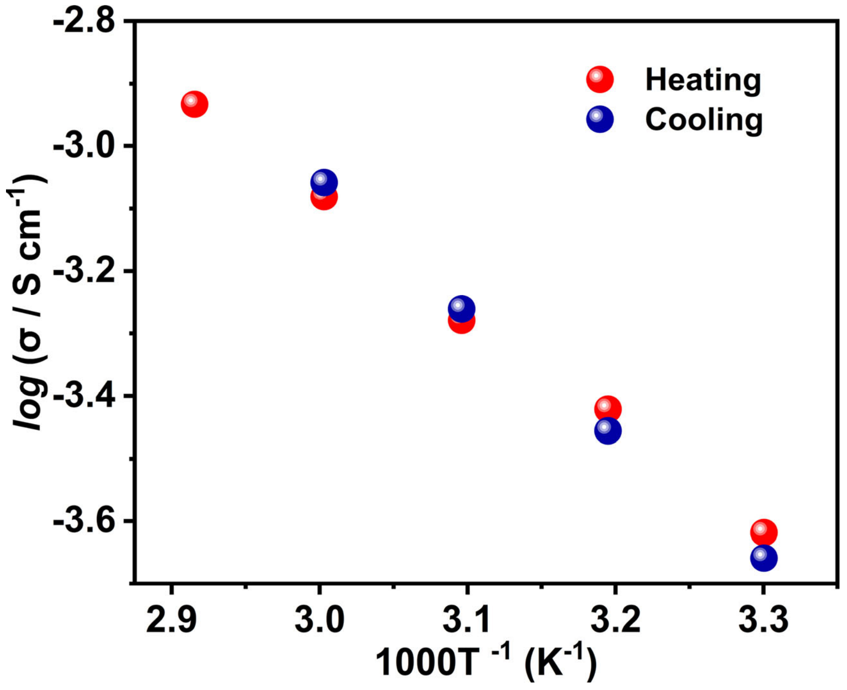

Supplementary Figure 37. Temperature-cycle proton conductivity of 1. Arrhenius plots of the proton conductivity of **1** during one heating and cooling cycle at 98% RH.

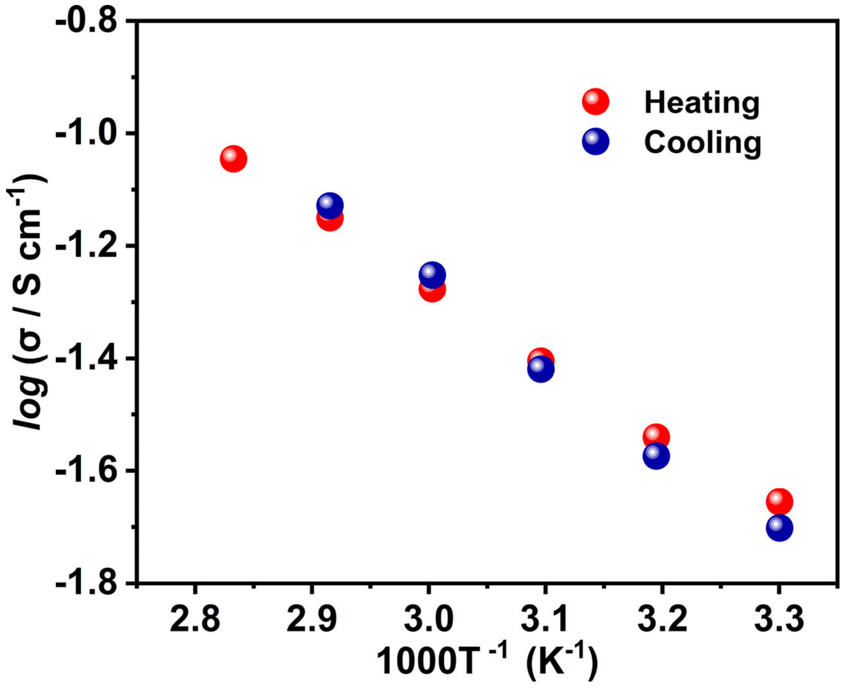

Supplementary Figure 38. Temperature-cycle proton conductivity of 2. Arrhenius plots of the proton conductivity of **2** during one heating and cooling cycle at 98% RH.

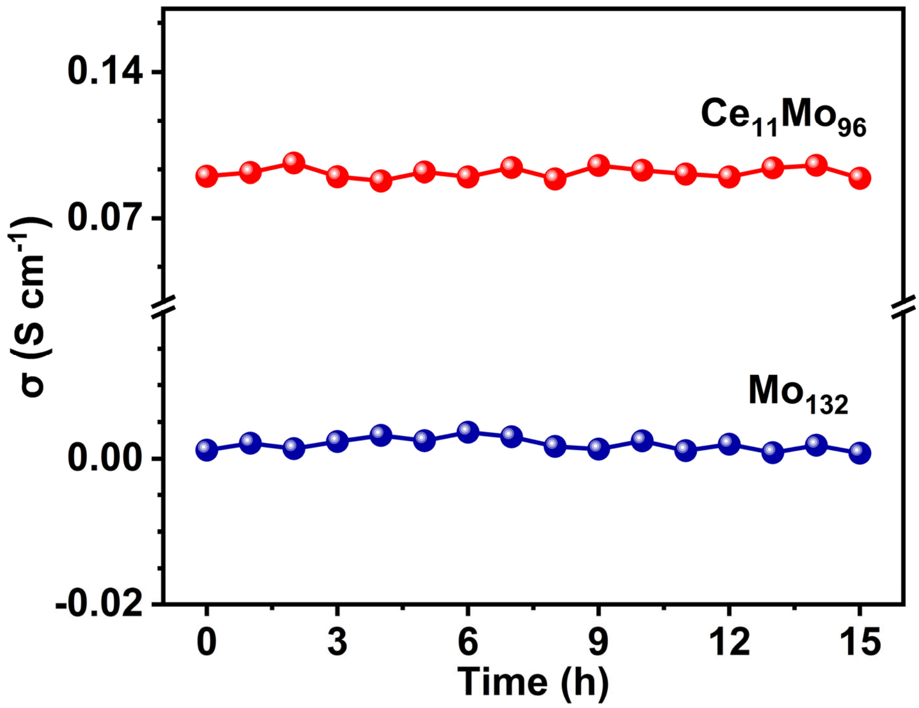

Supplementary Figure 39. Time-dependent proton conductivity. Time-dependent bulk conductivity of **1** (70°C) and **2** (80°C) measured at 98% RH.

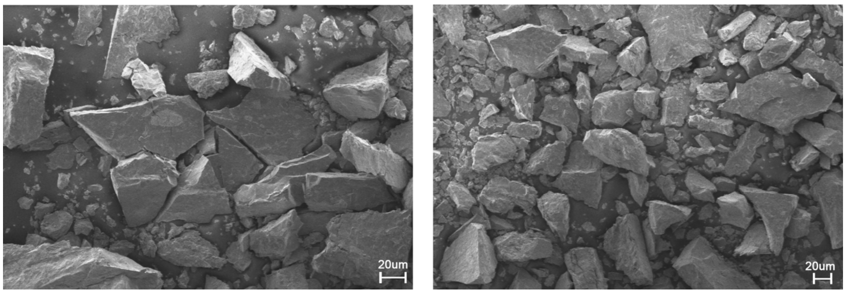

Supplementary Figure 40. SEM images of sample morphology. SEM for **2** before (left) and after (right) proton-conductive measurement.

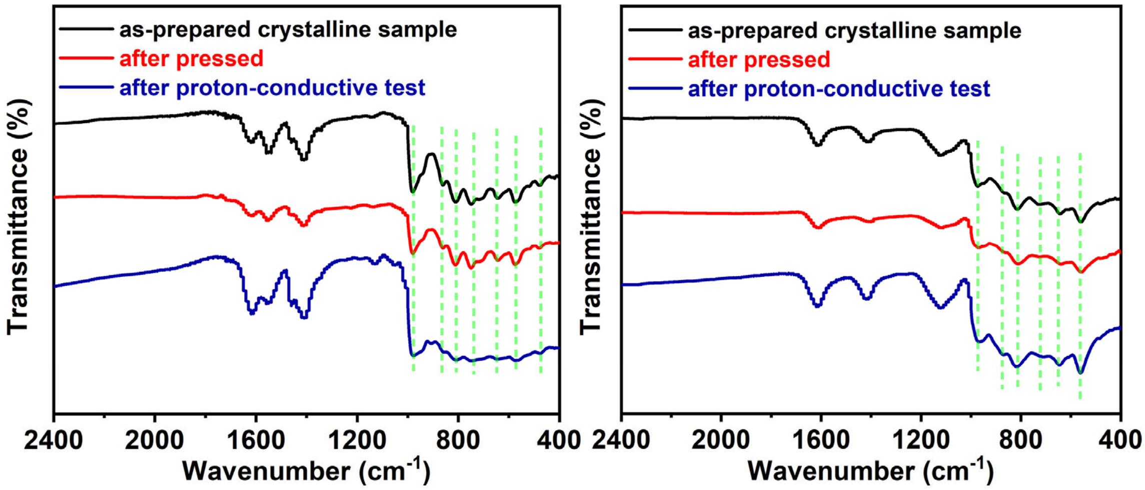

Supplementary Figure 41. FT-IR spectra of 1-2. FT-IR spectra of **1** (left) and **2** (right) with the samples after been pressed into pellet and after proton-conductive measurement.

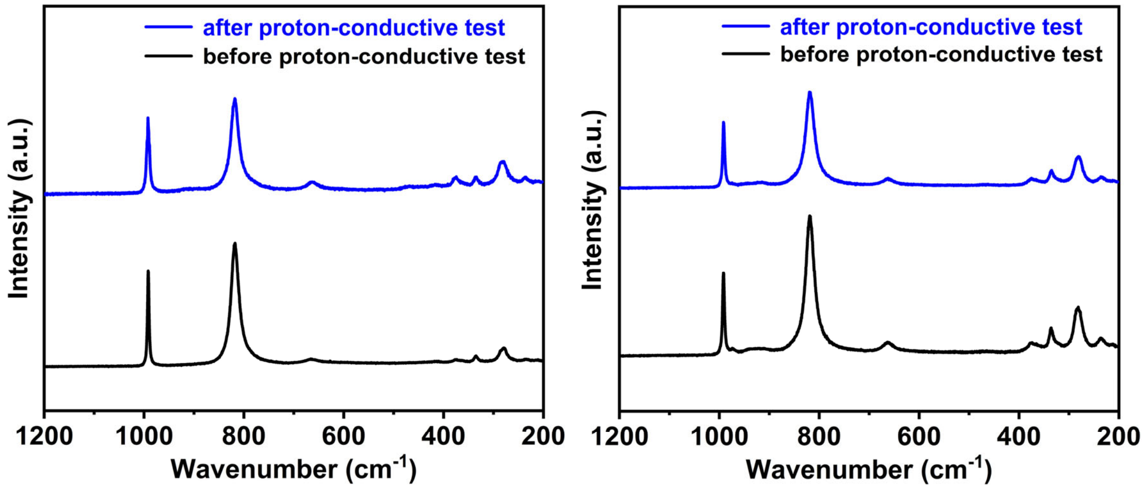

Supplementary Figure 42. Raman spectra for 1-2. Raman spectra for **1** (left) and **2** (right) before and after proton-conductive measurement.

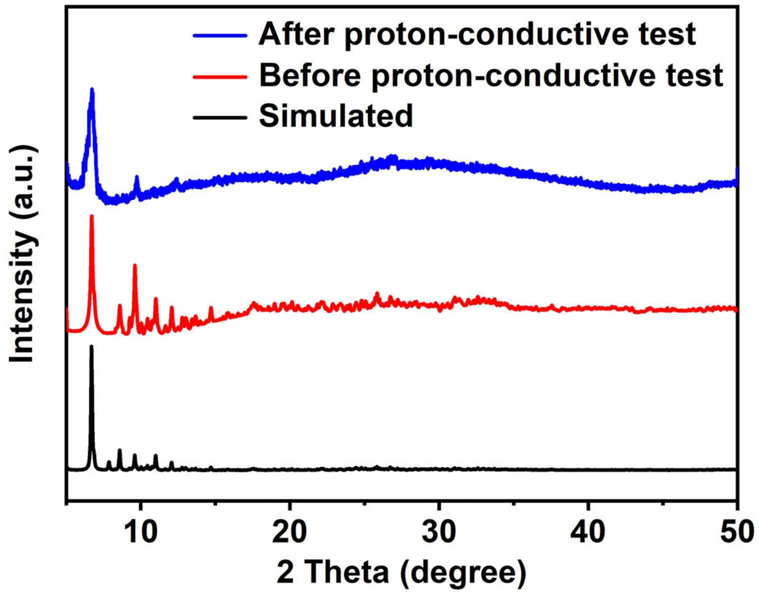

Supplementary Figure 43. PXRD for 1. PXRD of **1**, calculated (black), crystalline sample (red), and after the proton-conductive measurement (blue).

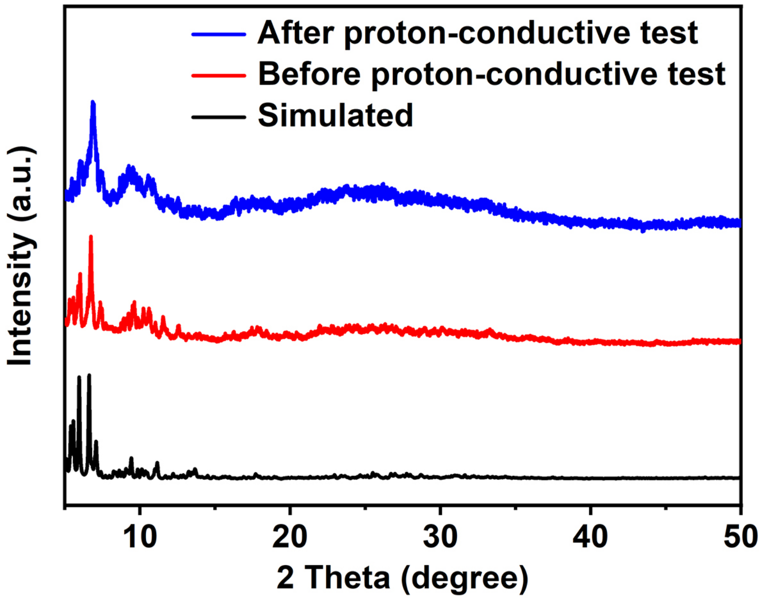

Supplementary Figure 44. PXRD for 2. PXRD of **2**, calculated (black), crystalline sample (red), and after the proton-conductive measurement (blue).

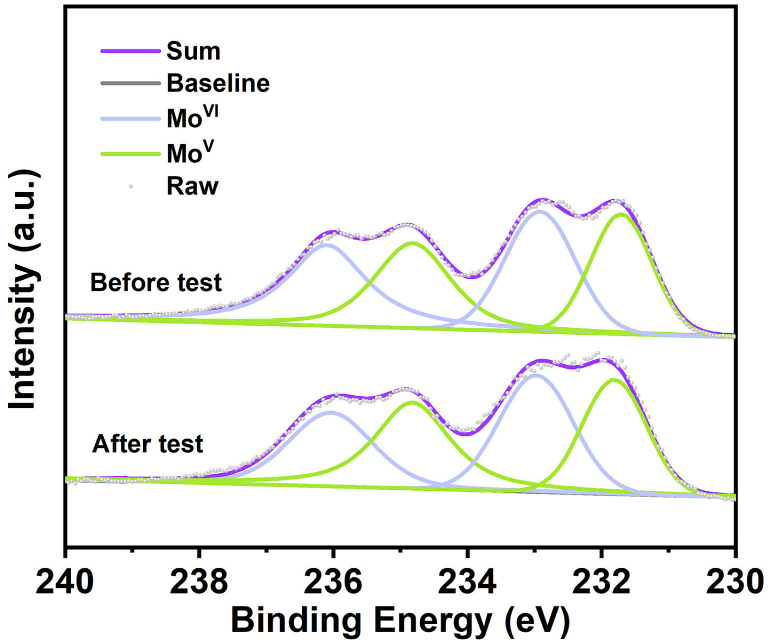

Supplementary Figure 45. XPS spectra of Mo. XPS spectra of Mo in **1** before and after proton-conductive measurement.

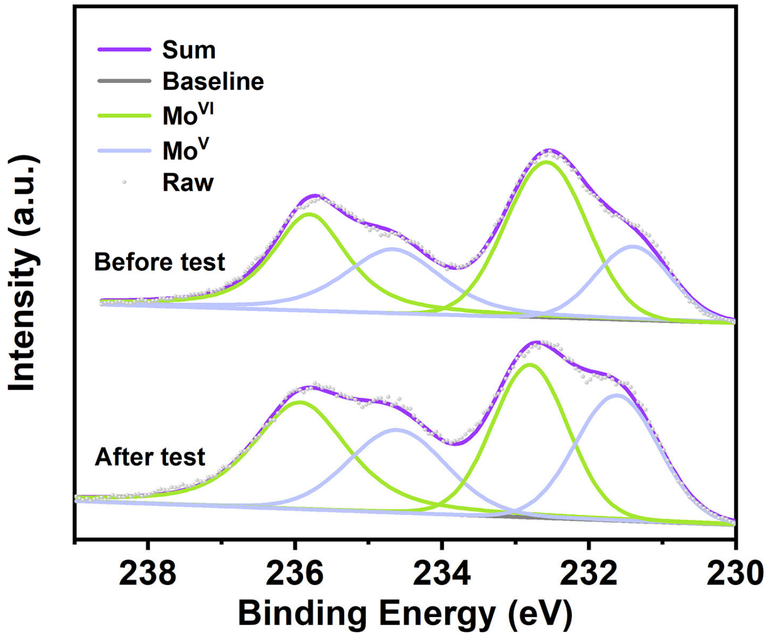

Supplementary Figure 46. XPS spectra of Mo. XPS spectra of Mo in **2** before and after proton-conductive measurement.

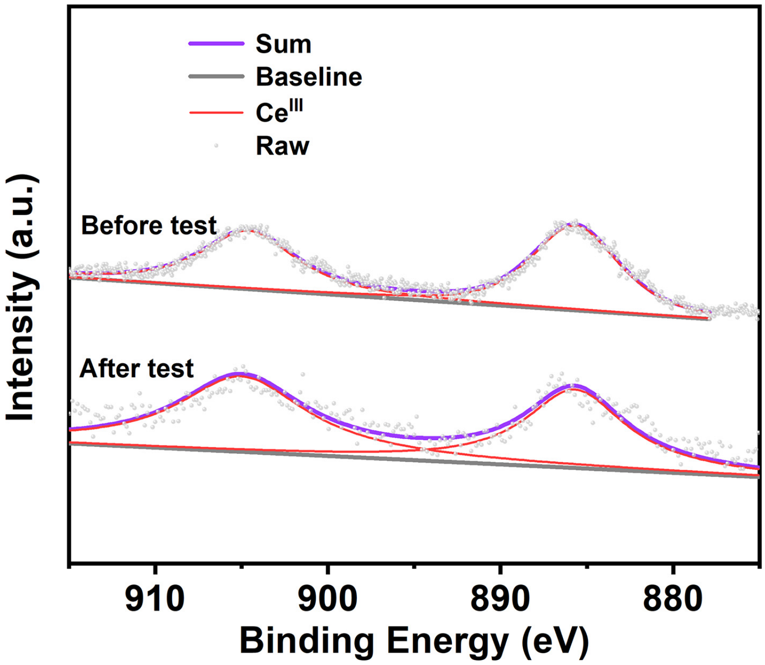

Supplementary Figure 47. XPS spectra of Ce. XPS spectra of Ce in **2** before and after proton-conductive measurement.

Supplementary Table 11. A comparison of the proton conductivity of **1** and **2** and some other recent representative POMs-based crystalline conducting materials.

Year	POMs-based crystalline conducting materials	Proton conductivity (S cm^{-1})	Relative Humidity	Temperature (°C)	E_a (eV)	*Reference*
2023	$[Mo_{132}O_{372}(OH)_{10}(H_2O)_{12}(SO_4)_5(CH_3COO)_{20}]^{52-}$	1.17 × 10^{-3}	98%	70	0.38	*This work*
	$[Ce_{11}Mo_{96}O_{286}(H_2O)_{101}(SO_4)_8]^{9-}$	2.21 × 10^{-2}	98%	30	0.29	
		0.90 × 10^{-1}	98%	80		
2022	$[Cu(en)_2(H_2O)]_2[Cu(en)_2]_{10}H_{97}[Dy_{10}Nb_{190}]^{7-}$	1.19 × 10^{-4}	98%	25	0.54	1
		3.75 × 10^{-3}	98%	85		
2022	$[(AsW_9O_{33})_6\{W_2O_5(H_2O)(Ala)\}_2\{W_3O_6(H_2O)(Ala)\}_2\{W_2O_5(Ala)\}]$	2.83 × 10^{-4}	75%	65	0.54	2
2022	$H_4[Cu(en)_2]_4\{K_4(H_2O)_2[Cu(en)_2]_5[Cu_5(trz)_2(en)_4(OH)_2][Dy_2Cu_2(en)_2(CO_3)_3(H_2O)_2(OH)_3][Dy(H_2O)_4][DyNb_{23}O_{68}(H_2O)_4]_2\}\cdot 60H_2O$	4.68 × 10^{-6}	98%	25	1.03	3
		3.42 × 10^{-3}	98%	85		
2022	$[(NaP_5W_{30}O_{110})_2\subset Mo_{22}(Fe\text{-}edta)_8O_{68}(H_2O)_2]^{40-}$	1.7 × 10^{-2}	90%	95	0.31	4
2022	$[\{Mo_{24}O_{48}(OMe)_{32}\}\{Mo_{24}O_{52}(OMe)_{28}\}_2]^{8-}$	6.73 × 10^{-6}	98%	35	0.49	5
		1.79 × 10^{-3}	98%	85		
2022	$H\{Ln_4(L)_2(H_2O)_{21}[Zr_3(OH)_3(PW_9O_{34})_2]\}\cdot 15H_2O$	7.53 × 10^{-3}	98%	85	0.30	6
2022	$\{[Co(en)_2(SO_3)][Te_4Nb_{24}O_{79}]\}^{20-}$	8.13 × 10^{-5}	75%	25	0.28	7
		3.05 × 10^{-4}	75%	60		
2021	$\{[Cu(en)_2]_{10}[Nb_{68}O_{182}(OH)_8(H_2O)_{10}]\}^{12-}$	9.67 × 10^{-5}	98%	25	0.53	8
		5.71 × 10^{-3}	98%	75		
2021	$[Co(H_2O)_6]_2\{[Co(H_2O)_4]_4[WZn_3(H_2O)_2(ZnW_9O_{34})_2]\}$	3.55 × 10^{-4}	98%	85	0.24	9
2021	$\{[P_2W_{15}Nb_3O_{62}]_2(4PBA)_2((4PBA)_2O)\}^{16-}$	1.64 × 10^{-3}	98%	20	0.66	10
2020	$[Mo^{V}_{180}Mo^{VI}_{60}(OH)_{60}O_{620-x}(SO_3)_{20-x}(SO_4)_x]^{-(80-2x)}$	3.3 × 10^{-2}	98%	25	0.24	11
		1.03 × 10^{-1}	98%	80		
2019	$[Mo_{72}^{VI}Mo_{60}^{V}O_{372}(CH_3COO)_{30}(H_2O)_{72}]^{44-}$	6.2 × 10^{-3}	98%	25	0.51	12
		5.0 × 10^{-2}	98%	60		
2018	$\{[W_{14}Ce^{IV}_6O_{61}]([W_3Bi_6Ce^{III}_3(H_2O)_3O_{14}][BiW_9O_{33}]_3)_2\}^{34-}$	2.4 × 10^{-3}	90%	25	0.68	13
2018	$\{[Na(NO_3)(H_2O)]_4[Al_{16}(OH)_{24}(H_2O)_8(P_8W_{48}O_{184})]\}^{16-}$	9.1 × 10^{-3}	85%	25	0.32	14
		4.5 × 10^{-2}	70%	85		
2018	$[Na_6(H_2O)_{12}]_4[K_{42}Ge_8W_{72}O_{272}(H_2O)_{60}]^{14-}$	3.3 × 10^{-3}	98%	30	0.52	15

		6.8×10^{-2}	98%	80		
2017	$[La_{27}Ge_{10}W_{106}O_{406}(OH)_4(H_2O)_{24}]^{59-}$	4.0×10^{-5}	98%	30	0.42	16
		1.5×10^{-2}	98%	85		
2016	$[Ce^{III}(H_2O)_6]\{[Ce^{IV}{}_7Ce^{III}{}_3O_6(OH)_6(CO_3)(H_2O)_{11}][(P_2W_{16}O_{59})]_3\}^{16-}$	1.95×10^{-7}	75%	30	0.36	17
		2.65×10^{-4}	75%	100		

Supplementary References

[1] R.-D. Lai. et al. Assemblies of increasingly large Ln-containing polyoxoniobates and intermolecular aggregation–disaggregation Interconversions. *J. Am. Chem. Soc.* **144**, 42-45(2022).

[2] K. Zheng. et al. dl-Alanine covalently bonded giant arsenotungstate with rapid photochromic and decent proton conduction properties. *Inorg. Chem.* **61**, 20222–20226(2022).

[3] R.-D. Lai.et al. Proton-conductive polyoxometalate architectures constructed from lanthanide-incorporated polyoxoniobate cages. *Inorg. Chem.* **61**, 21047–21054.(2022)

[4] M. Zhu. et al. Macrocyclic polyoxometalates: selective polyanion binding and ultrahigh proton conduction. *Angew. Chem. Int. Ed.* **61**, e202200666(2022).

[5] Y. Wang. et al. A high-nuclear isopolymolybdate cluster assembled with an anionic $[\{Mo_{24}O_{48}(OMe)_{32}\}]^{8-}$ and two charge-neutral $[\{Mo_{24}O_{52}(OMe)_{28}\}]$ cages. *Chem. Eur. J.* **28**, e202200637(2022).

[6] Y.-H. Fan. et al. Construction of water-stable rare-earth organic frameworks with ambient high proton conductivity based on zirconium sandwiched heteropolytungstate. *Inorg. Chem.* **61**, 13829–13835(2022).

[7] Z. Jing. et al. Rocket-shaped telluroniobate with efficient catalytic activity in the transesterification reaction. *Inorg. Chem.* **61**, 16528–16532(2022).

[8] Y.-D. Lin. et al. Proton conductive polyoxoniobate frameworks constructed from nanoscale $\{Nb_{68}O_{200}\}$ cages. *Chem. Commun.* **57**, 4702–4705(2021).

[9] C. Si. et al. A polyoxometalate-based inorganic porous material with both proton and electron conductivity by light actuation: photocatalysis for baeyer–villiger oxidation and Cr(VI) reduction. *Inorg. Chem.* **60**, 682–691(2021).

[10] S. Li. et al. High proton-conductivity in covalently Linked polyoxometalate-organoboronic acid-polymers. *Angew. Chem. Int. Ed.* **60**, 16953–16957(2021).

[11] J. Lin. et al. Self-assembly of giant Mo_{240} hollow opening dodecahedra. *J. Am. Chem. Soc.* **142**, 13982−13988(2020).

[12] W.-J. Liu.et al. Different protonic species affecting proton conductivity in hollow spherelike polyoxometalates. *ACS Appl. Mater. Interfaces.* **11**, 7030−7036(2019).

[13] J.-C. Liu. et al. Aggregation of giant cerium–bismuth tungstate clusters into a 3D porous framework with high proton conductivity. *Angew. Chem. Int. Ed.* **57**, 8416–8420(2018).

[14] P. Yang. et al. Lewis acid guests in a $\{P_8W_{48}\}$ archetypal polyoxotungstate host: enhanced proton conductivity via metal-oxo cluster within cluster assemblies. *Angew. Chem. Int. Ed.* **57**, 13046–13051(2018).

[15] Z. Li. et al. All-inorganic ionic porous material based on giant spherical polyoxometalates containing core-Shell $K_6@K_{36}$-water cage. *Angew. Chem. Int. Ed.* **57**, 15777–15781(2018).

[16] Z. Li. et al. Four-shell polyoxometalates featuring high-nuclearity Ln_{26} clusters: structural transformations of nanoclusters into frameworks triggered by transition-metal Ions. *Angew. Chem. Int. Ed.* **56**, 2664–2669(2017).

[17] P. Ma. et al. Coordination-driven self-assembly of a 2D graphite-like framework constructed from high-nuclear Ce_{10} cluster encapsulated polyoxotungstates. *Inorg. Chem.* ***55***, 918–924(2016).
